# A novel strategy to enhance terpenoids production using cambial meristematic cells of *Tripterygium wilfordii* Hook. f.

**DOI:** 10.1186/s13007-019-0513-x

**Published:** 2019-11-07

**Authors:** Yadi Song, Shang Chen, Xiujuan Wang, Rui Zhang, Lichan Tu, Tianyuan Hu, Xihong Liu, Yifeng Zhang, Luqi Huang, Wei Gao

**Affiliations:** 1 School of Traditional Chinese Medicine, Capital Medical University, Beijing, 100069 China; 2 School of Pharmaceutical Sciences, Capital Medical University, Beijing, 100069 China; 3 State Key Laboratory of Dao-di Herbs, National Resource Center for Chinese Materia Medica, China Academy of Chinese Medical Sciences, Beijing, 100700 China; 4 Advanced Innovation Center for Human Brain Protection, Capital Medical University, Beijing, 100069 China

**Keywords:** Cambial meristematic cells, *Tripterygium wilfordii* Hook. f, Triptolide, Celastrol, Triptophenolide

## Abstract

**Background:**

*Tripterygium wilfordii* Hook. f. (*T. wilfordii*) is an important medicinal plant with anti-inflammatory, immunosuppressive and anti-tumor activities. The main bioactive ingredients are diterpenoids and triterpenoids, such as triptolide, triptophenolide and celastrol. However, the production of terpenoids from original plants, hairy roots and dedifferentiated cells (DDCs) are not satisfactory for clinical applications. To find a new way to further improve the production of terpenoids, we established a new culture system of cambial meristematic cells (CMCs) with stem cell-like properties, which had strong vigor and high efficiency to produce large amounts of terpenoids of *T. wilfordii*.

**Results:**

CMCs of *T. wilfordii* were isolated and cultured for the first time. CMCs were characterized consistent with stem cell identities based on their physiological and molecular analysis, including morphology of CMCs, hypersensitivity to zeocin, thin cell wall and orthogonal partial least square-discriminant analysis, combination of transcriptional data analysis. After induction with methyl jasmonate (MJ), the maximal production of triptolide, celastrol and triptophenolide in CMCs was 312%, 400% and 327% higher than that of control group, respectively. As for medium, MJ-induced CMCs secreted 231% triptolide and 130% triptophenolide at the maximum level into medium higher than that of control group. Maximal celastrol production of induced CMCs medium was 48% lower than that of control group. Long-term induction significantly enhanced the production of terpenoids both in cells and medium. The reason for increasing the yield of terpenoids was that expression levels of *1*-*deoxy*-d-*xylulose*-*5*-*phosphate synthase* (*DXS*), *1*-*deoxy*-d-*xylulose*-*5*-*phosphate reductoisomerase* (*DXR*) and *hydroxymethylglutaryl*-*CoA synthase* (*HMGS*) were upregulated in CMCs after induction.

**Conclusions:**

For the first time, CMCs of *T. wilfordii* were isolated, cultured, characterized and applied. Considering the significant enrichment of terpenoids in CMCs of *T. wilfordii*, CMCs could provide an efficient and controllable platform for sustainable production of terpenoids, which can be a better choice than DDCs.

## Background

Plants are an important source for producing a vast reservoir of natural products and a common strategy for obtaining natural products is to extract from original plants, but typically the resources and the growth rate of plants limit the yields of natural products [1, 2]. *Tripterygium wilfordii* Hook. f. (*T. wilfordii*) is a medicinal plant of Celastraceae family, whose dried root is a well-known traditional Chinese medicine with a long history for treating inflammation, tumor, immune regulation, human immunodeficiency virus (HIV) and Parkinson’s disease, having aroused great interest in the field of medicine [3–5]. Generally, the roots of 6-year-old *T. wilfordii* were selected as Chinese herbal medicine. Terpenoids are major bioactive components responsible for pharmacological effects of *T. wilfordii* including triptolide, celastrol and triptophenolide, from which, triptolide is a diterpenoid epoxide known for anti-inflammatory and anti-cancer activities [6–9] and it has been applied in Phase I clinical trials of prostate cancer [10]. Celastrol is a pentacyclic triterpenoid with the effect of treating obesity, metabolic syndrome and tumor [11, 12]. Triptophenolide is an abietane diterpenoid and identified as a therapy for treating cancers, such as prostate cancer [13]. Triptolide and celastrol are considered to be key bioactive compounds for clinical applications isolated from traditional medicinal plants [14]. However, the main challenge in the application of *T. wilfordii* is the insufficient supply of triptolide, celastrol and triptophenolide due to the yield of them in plants is poor and alternative along with long period of growth [15].

Although a little work of tissue culture and cells culture has been done to produce terpenoids of *T. wilfordii*, which is important to reduce dependence on natural resources, the production of terpenoids from hairy roots [16, 17] and suspension cells [18] are also unsatisfactory. An alternative to solve above problems is needed. Currently, plant suspension cell culture is becoming a well-established platform for the biosynthesis of natural products, especially for the natural products with high economic value or complex molecules [19]. Plant suspension cells derived from dedifferentiated cells (DDCs) which typically exhibit cellular heterogeneity [20] and variability in natural products yields [21]. A major breakthrough in plant cell culture for the production of natural products was the isolation of cambial meristematic cells (CMCs) [22]. Only a few cells which exist in meristems located at the tips of shoots, at the tips of roots or within the vascular tissue can actively divide in plants, giving rise to a variety of cells that eventually undergo a differentiation process and simultaneously produce new stem cells [23]. CMCs are essentially undifferentiated cells with plant stem cells properties [24] and they can accumulate natural products stably for a long period [22]. So far, several successful cases of obtaining plant CMCs have been reported [22, 25–27]. However, CMCs have rarely been used in recent years, especially in the acquisition of natural products.

In this work, we isolated and characterized CMCs of *T. wilfordii* for the first time and they were identified by their morphology, hypersensitivity to radiomimetic drug zeocin, thin cell wall, orthogonal partial least square-discriminant analysis and transcriptional data analysis. Moreover, the difference of metabolites of CMCs and DDCs were detected by ultra-performance liquid chromatography-quadrupole time-of-flight mass spectrometry (UPLC/Q-TOF MS). In addition, the ability of CMCs to produce triptolide, celastrol and triptophenolide was evaluated after induction with methyl jasmonate (MJ) as well as after long-term induction culture of MJ. Then the relative expression levels of some key enzyme genes in terpenoids biosynthetic pathway were investigated using quantitative real-time reverse transcription-polymerase chain reaction (qRT-PCR). Through isolation, culture, identification, transcriptome and metabolites data analysis, combined with exogenous induction and long-term induction, we obtained CMCs with stem cell-like properties and strong vigor, which provided a novel strategy to enhance the production of terpenoids in *T. wilfordii*.

## Results

### Isolation of CMCs and DDCs

**Fig. 1:**
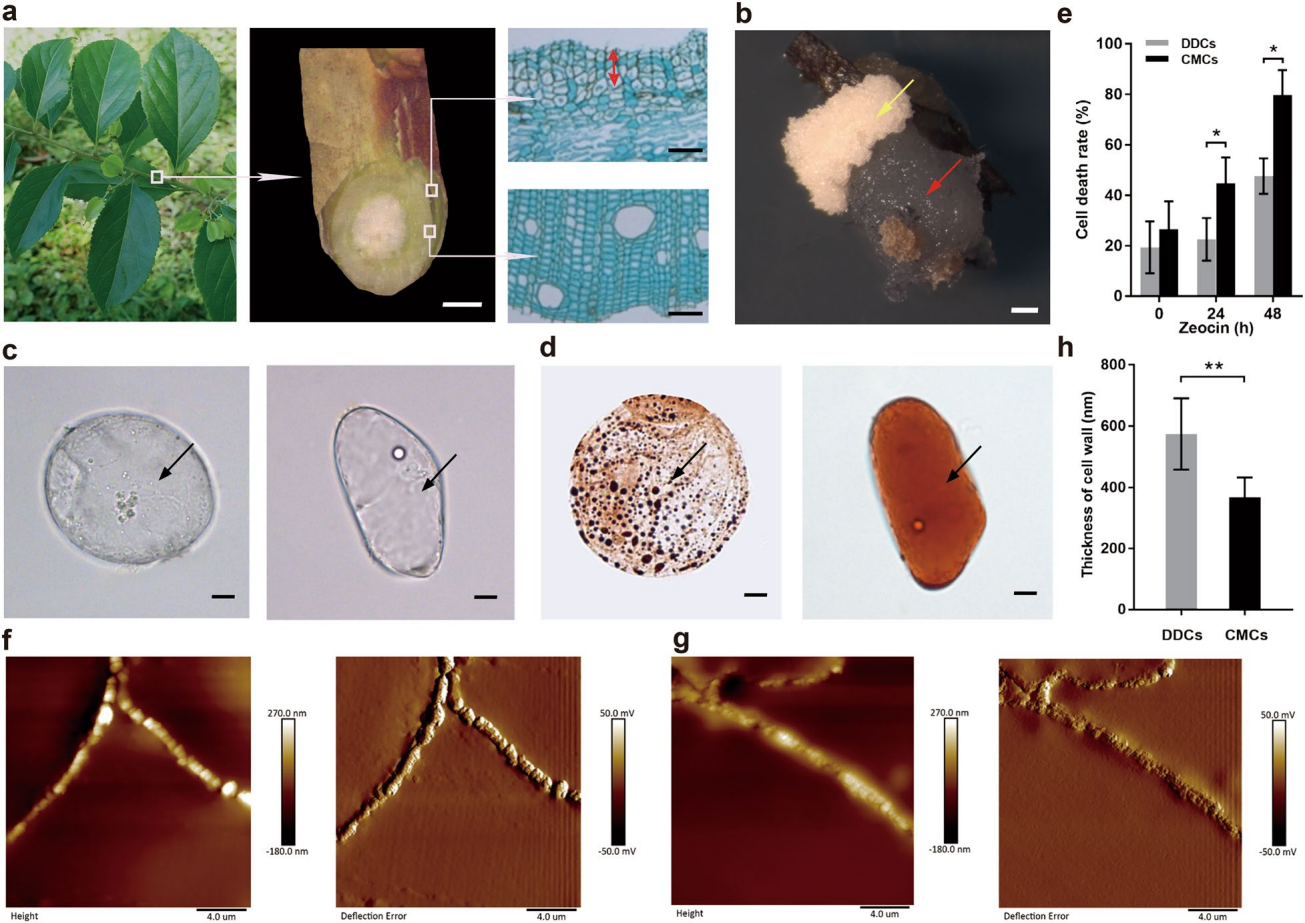
The isolation and characteristic of CMCs and DDCs from *T. wilfordii*. **a** Preparation of *T*. *wilfordii* by peeling off cambium, phloem, cortex and epidermal tissue from the xylem and the epidermal tissue side. Cross-section of cambium cell layers (orange arrow) along with phloem tissue. The bottom micrograph shows the xylem. Cells were stained by fast green. White scale bar, 0.25 mm. Black scale bar 10 μm. **b** A visible natural split of CMCs (red arrow) from DDCs (yellow arrow). Scale bar,1 mm. **c** Micrographs of CMCs and DDCs. CMCs possess plentiful and small vacuoles, one of which is marked by a black arrow. DDCs only have one large vacuole denoted by a black arrow. Scale bar, 10 μm. **d** Single CMC and DDC stained with neutral red. The vacuole stained red is indicated by a black arrow. Scale bar, 10 μm. **e** Effects of zeocin on the death of CMCs and DDCs. Data points represent the mean ± standard deviations (*SD*), n = 3, *P < 0.05. **f** Surface morphology of CMC walls observed by AFM. Height image and deflection error image. Scale bar, 4 μm. **g** Surface morphology of DDC walls observed by AFM. Height image and deflection error image. Scale bar, 4 μm. **h** The thickness of CMC and DDC walls. Values are expressed as mean ± SD, n = 30, **P < 0.01

The stems were collected from wild *T. wilfordii*. We removed the xylem and pith and cultured the tissue that contained cambium, phloem, cortex and epidermis on the solid isolated medium (Fig. 1a). Lignin deposition was detected by staining with phloroglucinol-HCl to confirm the complete removal of xylem tissue (Additional file 1: Figure S1). There was a visible split between prospective CMCs and DDCs after cultured for 30 d. Dim, soft and proliferating prospective CMCs could be easily separated from the white and irregular DDCs (Fig. 1b). CMCs and DDCs were cultured in Murashige & Skoog (MS) liquid medium containing 2 mg L^−1^ 2,4-dichlorophenoxyacetic acid (2,4-D), 2 mg L^−1^ naphthalene-acetic acid (NAA), 30 g L^−1^ sucrose.

### Characteristic identification of CMCs and DDCs

Through the observation under a light microscope, it revealed that single cell derived from cambium of stem possessed abundant and small vacuoles, which is a characteristic feature of CMCs [28]. Compared with that, single DDC had only one large vacuole (Fig. 1c), which is the distinct trait of this kind of cells. The difference above was more noticeable when vacuoles were stained with neutral red (Fig. 1d).

Incubated with 200 μg mL^−1^ radiomimetic drug zeocin for different times, the cell death rate of these cultured cells gradually increased as incubation time rose (Fig. 1e). Moreover, prospective CMCs had a death rate of 79.8% after incubation for 48 h, higher than that of DDCs, which was 47.6%. These results indicated prospective CMCs were peculiarly hypersensitive to the radiomimetic drug zeocin, while DDCs were not, which indicated that the cells we cultured were CMCs [22].

### Thinner cambial meristematic cell walls

To establish whether CMC walls are thinner than that of DDC walls [29], adding a new approach for CMCs identification, we first explored the performance of their walls at nanoscale levels using atomic force microscope (AFM), which could not only investigate morphological characteristics of cell walls but also measure the thickness of them. CMC walls consisted of numerous small raised nodules, whose boundaries were clear inside cells and the height of CMC walls were much higher than that of DDCs (Fig. 1f). In contrast, the surface roughness of DDC walls were more noticeable. Moreover, the raised nodules were smaller and their edges were diffusing inside cells (Fig. 1g). In addition, the wall of each kind of cell had a non-uniform thickness, especially at the interface of adjoining cells. The measurement result showed that CMC walls and DDC walls had average thicknesses of 367.9 ± 64.27 nm and 573.7 ± 116.5 nm, respectively (Fig. 1h) and the thicknesses of CMC walls were significantly smaller than that of DDC walls, marking that CMC walls was thinner than DDC walls, which may be another reason for the lower shear stress of CMCs.

### Comparative transcriptome analysis

We used RNA Sequencing (RNA-seq) technology to compare the characteristics of CMCs and DDCs at the molecular level. After removal of adapter and low-quality sequences, 76.08 million high quality reads totaling 11.39 Gb were obtained. The clean reads were assembled using the Trinity software [30] to obtain the de novo reference sequence. A total of 335,609 transcripts of average length 1033 bp of sequence were obtained (Additional file 1: Figure S2a). Depended on these data, we used Corset software to summarize read counts to clusters (maximum length, 17,305 bp; average length, 1241 bp), from which, 260,930 unigenes were constructed, with the average length of 1241 bp and N50 of 1712 bp (Additional file 1: Table S1). Unigenes were used for further analysis. All unigenes were annotated against the National Center for Biotechnology Information (NCBI) non-redundant (Nr) protein database, NCBI nucleotide (Nt) database, Protein family (Pfam) database, the Eukaryotic ortholog groups (KOG) database, the Swiss-Prot protein database, Gene Ontology (GO) database and Kyoto Encyclopedia of Genes and Genomes Orthology (KO) database to predict the protein functions by annotations of the most similar proteins. A total of 32,444 unigenes were annotated (about 12.43% of all assembled unigenes) in the seven public databases (Additional file 1: Figure S2b) and 213,306 unigenes (81.74%) were annotated in at least one database.

**Fig. 2:**
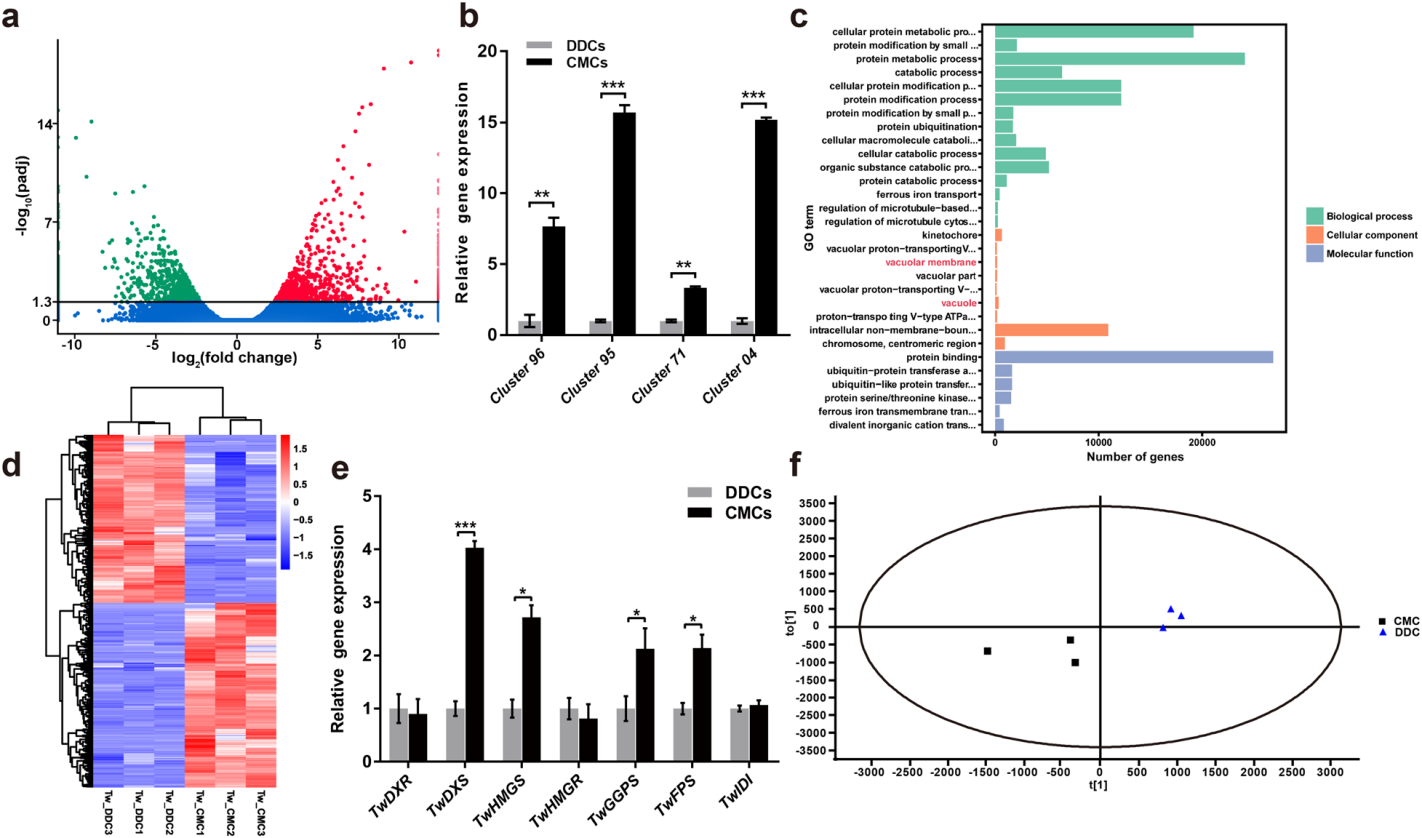
Transcriptome data and OPLS-DA analysis of CMCs and DDCs from *T. wilfordii*. **a** Scattered plot of DEGs analysis between CMCs and DDCs. Genes with red color were up-regulated and genes with green color were down-regulated, blue plot indicates non-DEGs. At an adjusted *P* < 0.05. **b** Validation of Cluster 96, Cluster 95, Cluster 71 and Cluster 04 by qRT-PCR. Values are expressed as mean ± SD, n = 3, **P < 0.01 and ***P < 0.001. The expression level of relevant gene of DDC was used as an internal control. **c** GO classifications of CMC DEGs. Words in red color indicates genes related to vacuoles. **d** Heatmap of DEGs. The color bar indicates that log_10_(FPKM + 1) is from high to low. Red indicates up-regulated genes and blue denotes down-regulated genes for CMC or DDC samples shown in triplicate. **e** Relative expression analysis of *TwDXR*, *TwDXS*, *TwHMGS*, *TwHMGR*, *TwGGPPS*, *TwFPS* and *TwIDI* genes in the terpenoid biosynthetic pathway. Values are expressed as mean ± SD, n = 3, *P < 0.05 and ***P < 0.001. The expression level of relevant gene of DDC was used as an internal control. **f** OPLS-DA analysis of CMCs and DDCs. n = 3

Differential expression analysis showed that 1759 unigenes were differentially expressed in CMCs and DDCs, with 921 upregulated and 838 downregulated (Fig. 2a), from which some important differentially expressed genes (DEGs) were detected by qRT-PCR.

*Phloem intercalated with xylem* (*PXY*) encodes a leucine-rich repeat (LRR) receptor-like kinase (RLK), which can be a marker gene in CMCs [31]. One gene in DEGs named Cluster 96 exhibited high similarity to *PXY* (Additional file 1: Figure S3a) and was expressed differentially in our CMCs, which was 7.6 times higher than that in DDCs (Fig. 2b).

*WUSCHEL* (*WUS*) is the founding member of the *WUSCHELRELATED HOMEOBOX* (*WOX*) gene family and it encodes a transcription factor which involves in perpetuating stem cell activity and keeping differentiating cells [32]. One gene in DEGs named Cluster 95 exhibited high similarity to *WUS* (Additional file 1: Figure S3b) and its related genes. Gene expression analysis showed that this gene was upregulated 15-fold in CMCs relative to DDCs (Fig. 2b).

CLAVATA3 (CLV3) belongs to a family of 32 small proteins called CLAVATA3/EMBRYO SURROUNDING REGION-RELATED (CLE). *CLV3* gene promotes the progression of meristem cells toward organ initiation, which is produced by the shoot stem cells and diffuses to underlying cells to inhibit *WUS* in a negative feedback loop that regulates the size of the stem cell population [33]. One gene in DEGs named Cluster 71 exhibited high similarity to *CLV3* (Additional file 1: Figure S3c) and its expression level was 3 times in CMCs compared with DDCs (Fig. 2b).

The gibberellic acid (GA) 2-oxidase is a key enzyme regulating the flux of GA through deactivating biologically active GAs in plants and GAs levels were downregulated through direct upregulation of the GA degrading gene *GA 2*-*oxidase* [34]. One gene in DEGs named Cluster 04 was similar to *GA 2*-*oxidase* (Additional file 1: Figure S3d) and it was 15 times higher in expression level of CMCs than that of DDCs (Fig. 2b).

GO enrichment analysis was implemented to explore the molecular function, cellular component and biological process of our prospective CMCs. The result displayed that genes related to vacuoles were significantly enriched, which could demonstrate the microscopic characteristics of CMCs (Fig. 1c) in the molecular level (Fig. 2c). The heatmap showed cluster analysis of DEGs between prospective CMCs and DDCs (Fig. 2d), which revealed that CMCs and DDCs were remarkably different between each other. Collectively, it was further confirmed by our RNA-seq data that these cultured cells could be identified as CMCs and they had plant stem cell properties.

The relative expression levels of main enzyme genes related to terpenoids biosynthetic pathway were analyzed in CMCs and DDCs. 1-deoxy-d-xylulose-5-phosphate synthase (DXS), 1-deoxy-d-xylulose-5-phosphate reductoisomerase (DXR), hydroxymethylglutaryl-CoA synthase (HMGS), hydroxymethylglutaryl-CoA reductase (HMGR), isopentenyl diphosphate isomerase (IDI), geranylgeranyl diphosphate synthase (GGPS) and farnesyl diphosphate synthase (FPS) are important enzymes in terpenoid biosynthesis pathway [35, 36]. We analyzed genes of these key enzymes in *T. wilfordii* using specific qRT-PCR primers (Additional file 1: Table S2). The relative expression levels of *TwDXS*, *TwHMGS*, *TwGGPS* and *TwFPS* in CMCs were upregulated, which were 4.03, 2.72, 2.13 and 2.14 times greater than those of DDCs, respectively, whereas *TwDXR*, *TwHMGR* and *TwIDI* did not change significantly (Fig. 2e).

### Orthogonal partial least square-discriminant analysis for metabolites

Comparison of metabolites of CMCs and DDCs were analyzed by UPLC/Q-TOF MS and Progenesis QI software. Triptolide, celastrol and triptophenolide were confirmed in both cell lines (Additional file 1: Figures S4, S5, S6, S7, Additional file 1: Table S3). The principal component analysis (PCA) scores plot clearly showed separation of CMCs from DDCs when the data were plotted along axes defined by first and second principal components (PC, PC1, 74.18% and PC2, 11.07%). Orthogonal partial least square-discriminant analysis (OPLS-DA) [37] was performed to further compare and discriminate the two kinds of cells based on the UPLC/Q-TOF MS data of analytes (Fig. 2f). The variations in the various samples was 80% [R^2^Y(cum)] and predictive value of the models was 60% [Q^2^(cum)], which were more than 50%, indicating the model was good for analysis. Based on the above analysis, these cultured cells conformed to the characteristics of CMCs and they performed like plant stem cells.

### Production of terpenoids increased after induction with methyl jasmonate

**Fig. 3:**
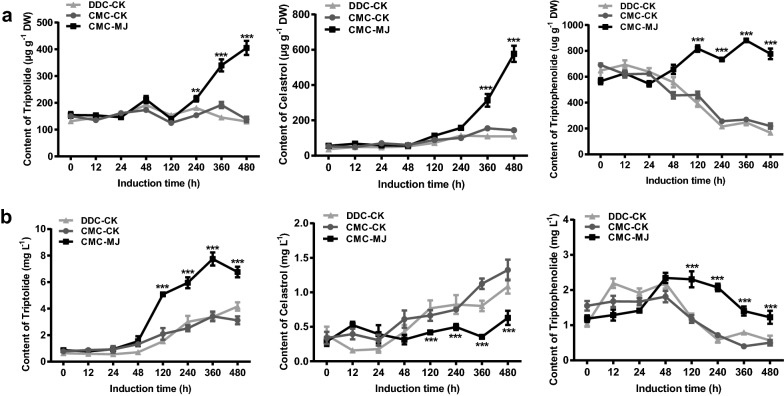
Terpenoids production of CMCs and DDCs from *T. wilfordii* following induction of 50 μmol L^−1^ MJ. **a** Total triptolide, celastrol and triptophenolide production of cells. **b** Total triptolide, celastrol and triptophenolide production of medium. The data represent the mean ± SD of five independent cell cultures. **P < 0.01 and ***P < 0.001. *DW* dry weight, *CK* control check, *MJ* methyl jasmonate

We then evaluated the ability of CMCs and DDCs which have cultured for 2 years in 100 mL Erlenmeyer flask to produce triptolide, celastrol and triptophenolide. At 10 d after subculture, elicitor methyl jasmonate (MJ) was added into the liquid medium with the final concentration 50 μmol L^−1^. Production of triptolide, celastrol and triptophenolide were measured at 0 h, 12 h, 24 h, 48 h, 120 h, 240 h, 360 h and 480 h after induction by ultra-performance liquid chromatography (UPLC) (Fig. 3, Additional file 1: Figure S8). The production of terpenoids in control checks (CK) group of CMCs and DDCs have little significant difference, while MJ induction group increased the yields of terpenoids relative to CK group. In terms of cells, the amount of triptolide produced in MJ-induced CMCs reached a peak of 405.1 μg g^−1^ at 480 h, which was 312% higher than that generated by CMCs in CK group (138.1 μg g^−1^) and by DDCs in CK group (130.4 μg g^−1^), and was 229% higher than that generated by MJ-induced DDCs (176.8 μg g^−1^) after the same induction time (Fig. 3a, Additional file 1: Figure S8a). Maximal production of celastrol (576.7 μg g^−1^) and triptophenolide (880.9 μg g^−1^) were obtained at 480 h and 360 h after MJ induction in CMCs, respectively, which corresponded to 400% and 327% of the respective yields in CK group of CMCs, 526% and 357% of those in CK group of DDCs. Meanwhile, MJ led a 373% increase on celastrol production, and 125% increase on triptophenolide production in MJ-induced CMCs compared to that of MJ-induced DDCs, respectively. As for medium, MJ-induced CMCs secreted 231% triptolide (7.75 mg L^−1^) at the maximum level into the medium at 360 h after induction, higher than the production of CMCs (3.42 mg L^−1^) and DDCs (3.36 mg L^1^) in CK group as well as DDCs (1.89 mg L^−1^) in MJ group (Fig. 3b, Additional file 1: Figure S8b). However, accumulation of celastrol in MJ-treated CMCs became lower than CK groups as induction time increased. Maximal celastrol production of CMCs was found on 480 h after treatment with MJ, at 0.63 mg L^−1^, was 48%, 58% lower than that of CMCs and DDCs in CK group, respectively, and was similar to that of DDCs in MJ group. For the production of triptophenolide in the medium, in MJ-induced CMCs, it started to rise at first, reaching a maximum (2.34 mg L^−1^) after 48 h of induction, and then it fell considerably, which had a 130%, 107% and 144% increase compared to that of CMCs in CK group, DDCs in CK group and DDCs in MJ group, respectively. Our findings implied that CMCs are dramatically more responsive to MJ induction and biosynthesize more terpenoids compared to DDCs of *T. wilfordii*.

### Relative expression analysis of genes involved in terpenoid biosynthesis after induction

**Fig. 4:**
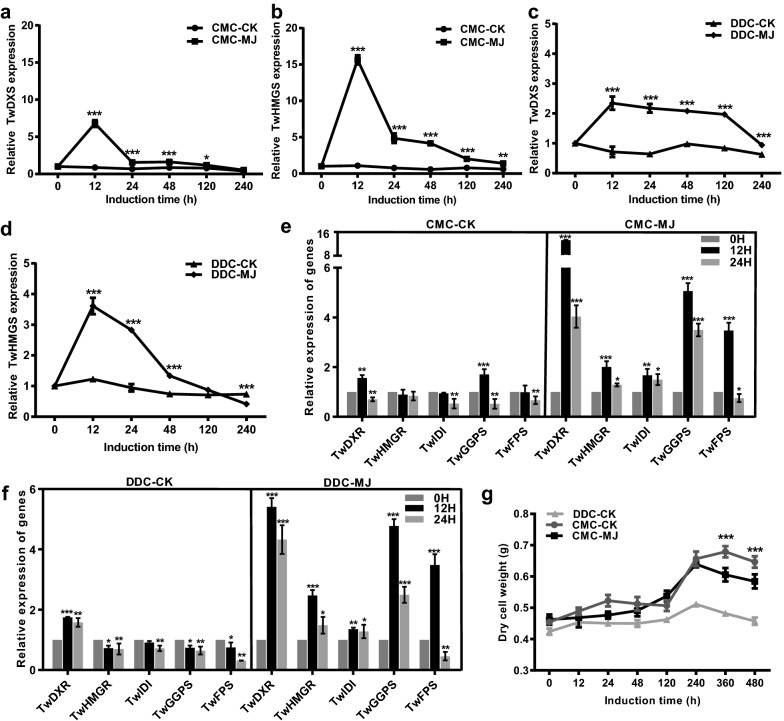
Effect of 50 μmol L^−1^-MJ on growth and expression of terpenoid biosynthetic genes in CMCs and DDCs. **a** Relative expression analysis of *TwDXS* in CMCs. **b** Relative expression analysis of *TwHMGS* in CMCs. **c** Relative expression analysis of *TwDXS* in DDCs. **d** Relative expression analysis of *TwHMGS* in DDCs. **e** Relative expression analysis of *TwDXR*, *TwHMGR*, *TwIDI*, *TwGGPPS* and *TwFPS* genes in the terpenoid biosynthetic pathway in CMCs. **f** Relative expression analysis of *TwDXR*, *TwHMGR*, *TwIDI*, *TwGGPPS* and *TwFPS* genes in the terpenoid biosynthetic pathway in DDCs. The expression level of 0 h as an internal control for **a**–**f**. **g** Growth of CMCs and DDCs follwing induction. Values represent mean ± SD, n = 4, *P < 0.05, **P < 0.01 and ***P < 0.001. *CK* control check, *MJ* methyl jasmonate

We next investigated the reasons for the higher yields of terpenoids in MJ-induced CMCs. The relative expression levels of *TwDXS* and *TwHMGS* were detected using qRT-PCR, which were significantly higher in CMCs than those of in DDCs in RNA-seq results (Fig. 2e). Maximal relative expression levels of *TwDXS* and *TwHMGS* occurred on 12 h after MJ induction in CMCs and were 6.78 and 15.6 times higher than those of CMCs in CK group, 2.95 and 4.33 times higher than those of DDCs in MJ group, respectively (Fig. 4a–d). Relative expression levels of other important genes involved in the terpenoids biosynthetic pathway including *TwDXR*, *TwHMGR*, *TwIDI*, *TwGGPS* and *TwFPS* were also measured. The maximal relative expression levels of *TwDXR*, *TwHMGR*, *TwIDI*, *TwGGPS* and *TwFPS* in MJ-induced CMCs were 9.36-, 2.25-, 1.77-, 2.98- and 3.53-fold greater than those of CMCs in CK after MJ induction for 12 h, respectively (Fig. 4e). In contrast to the relative expression level of *TwDXR* in MJ-induced CMCs increased by 2.71-fold compared to DDCs in MJ group after MJ induction for 12 h, the relative expression levels of *TwHMGR*, *TwIDI*, *TwGGPS* and *TwFPS* were not significantly affected (Fig. 4e, f).

### The growth characteristics of CMCs

The growth characteristics of CMCs and DDCs were investigated after MJ induction by measuring dry cell weight. The weight of MJ-treated CMCs gradually increased to peak and then decreased as incubation time rose, grew much faster than DDCs (Fig. 4g, Additional file 1: Figure S9), but slower than CK group of CMCs. Growth of CMCs differ significantly between the CK and MJ-treated groups after 360 h of induction, indicating that MJ may had a negative effect on cell growth. Growth assessment showed that MJ-treated CMCs had generated a maximal dry weight (DW) of 0.64 g, an increase of 128% and 139% compared with DDCs in CK group and DDCs in MJ group after induction for 240 h, respectively, and was similar to that of CMCs in CK group. These results suggested that CMCs from *T. wilfordii* might provide a considerably better source for these terpenoids than DDCs.

### Long-term induction enhanced production of terpenoids

**Fig. 5:**
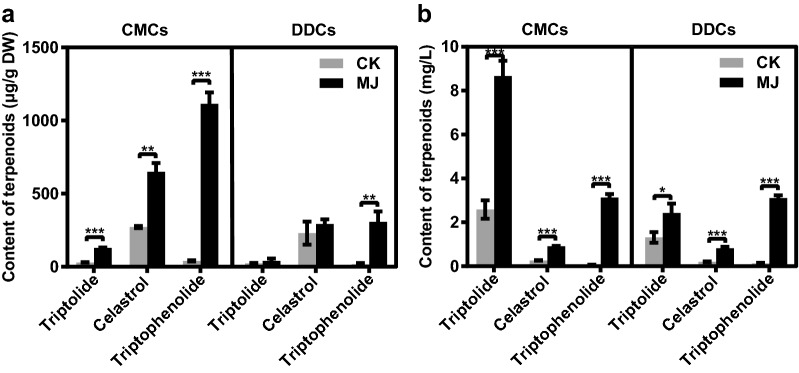
Long-term induction culture of *T. wilfordii* CMCs and DDCs with MJ. **a** Production of terpenoids in cells after long-term induction culture. **b** Production of terpenoids in medium after long-term induction culture. Values represent mean ± SD, n = 4, **P < 0.01 and ***P < 0.001. *CK* control check, *MJ* methyl jasmonate*, DW* dry weight

We determined the magnitude of accumulation of terpenoids in CMCs and DDCs of *T. wilfordii* induced by MJ for a long time. After 6 months of continuous induction culture in a 1000 mL Erlenmeyer flask containing 600 mL liquid medium, the amount of terpenoids produced by MJ-treated CMCs were 3.12 (triptolide), 2.23 (celastrol) and 3.63 (triptophenolide) times greater than that generated by MJ-treated DDCs, respectively (Fig. 5a). In addition, terpenoids were also secreted directly into the medium. MJ-treated CMCs secreted a strikingly greater amount of triptolide into the medium under these culture conditions than the corresponding DDCs (Fig. 5b).

## Discussion

### Two novel methods for identification of cambial meristematic cells

In this work, CMCs of *T. wilfordii* were isolated and cultured for the first time. The characteristic features of CMCs, including abundant and small vacuoles within each cell and hypersensitivity to zeocin, and the transcriptional data analysis were consistent with CMCs reported previously [22]. Additionally, it is the first time we proposed two novel methods that the thickness of CMC wall was thinner than that of DDC, as well as OPLS-DA analysis, to distinguish two types of cells through the difference of their cell wall thickness and cell metabolites based on quantitative analysis, which could be more accurate for identification of CMCs.

### Cambial meristematic cells produced terpenoids efficiently

Most bioactive terpenoids are present in low amounts in the *T. wilfordii* plant with the mean concentrations of 7.61, 53.83 and 179 μg g^−1^ for triptolide, celastrol and triptophenolide, respectively [38, 39]. Further, the complex structure of the pentacyclic triterpenoid celastrol highlights the complexity of plant extracts. After induction of CMCs of *T. wilfordii* in our work, UPLC results showed that the production of triptolide, celastrol and triptophenolide in CMCs was 53, 10 and 4.62 times higher than those of the *T. wilfordii* plant which has been reported previously, respectively [38, 39].

MJ is an abiotic elicitor that can be used efficiently to increase the production of terpenoids in *T. wilfordii*, especially for triptolide, celastrol and triptophenolide [40–42]. In our work, whether from cells or medium, we could produce specific terpenoid on a large scale based on different MJ induction times. Although the yield of terpenoids produced by CMCs was large in this work, there is still the possibility of improvement. The combination of elicitors is another common practice in increasing the production of terpenoids. The CMCs systems developed by Lee et al. produced much higher production of paclitaxel than that of corresponding DDCs systems after elicitors MJ, chitosan, together with a precursor phenylalanine were added into culture medium [22]. The selection and concentration of elicitors and the culture phase at the time of induction are all key factors necessary to optimize the production of terpenoids [22, 43, 44].

### Upregulation of genes in terpenoid biosynthetic pathways can increase yield of terpenoids

In plants, terpenoids are biosynthesized by the mevalonate (MVA) pathway located in the cytoplasm and the 2-C-methyl-d-erythritol-4-phosphate (MEP) pathway located in the plastid [36]. Isopentenyl pyrophosphate (IPP) can transfer through the plastid envelope to link the MEP and MVA pathways and IPP is the precursor of GGPP and farnesyl pyrophosphate (FPP) [45]. In the MEP pathway, GGPP produces triptophenolide and triptolide through a series of reactions. In the MVA pathway, FPP undergoes a series of reactions to form celastrol. MJ was previously shown to enhance expression levels of the terpenoids backbone biosynthetic genes *TwDXS*, *TwDXR* and *TwHMGS* in suspension cells of *T. wilfordii* [42, 46], but the effects of MJ on these genes in CMCs of *T. wilfordii* have not been studied. In our work, the results of relative expression analysis after induction with MJ suggested that the upregulation of *TwDXS*, *TwDXR* and *TwHMGS* expression levels led to an increase in the production of triptolide, celastrol and triptophenolide in CMCs of *T. wilfordii*. It is also indicated that the effects of *TwDXS*, *TwDXR* and *TwHMGS* on the biosynthesis of precursors IPP of the diterpenoids and triterpenoids in *T. wilfordii* may be consistent, which may be due to the interaction between genes [47]. Moreover, different downstream synthetic enzymes of terpenoids have different competing abilities for precursors, resulting in different levels of terpenoids accumulation. However, the key genes involved in downstream pathway of terpenoid biosynthesis in *T. wilfordii* have not been fully analyzed. The effects of genes from downstream pathways on the biosynthesis of terpenoids need to be further explored in CMCs of *T. wilfordii*.

The CMCs of *T. wilfordii* are expected to be a novel and powerful system for studying enzymes and pathways of terpenoid biosynthesis to enhance the production of the terpenoids through metabolic engineering [48, 49] and synthetic biology [50, 51] in the future.

## Conclusions

CMCs of *T. wilfordii* were isolated, cultured, characterized and applied for the first time. Considering the significant enrichment of terpenoids in CMCs of *T. wilfordii*, CMCs could provide an efficient and controllable platform for sustainable production of terpenoids, which can be a better choice than DDCs.

## Methods

### Collection and sterilization of *T. wilfordii* Samples

The stems of *T. wilfordii* plant were obtained from the Yongan national forest in Fujian Province, China. The stems collected were immediately washed with water, gently brush the surface with a soft brush and detergent, and rinsed with running tap water for 1 h, then soaked in distilled water for later use. Care should be taken to avoid mechanical damage to the explants during the cleaning process. After that, the surfaces were disinfected with 75% ethanol (added 2–3 drops of Tween-20) for 1 min, and rinsed 3 times with sterilized distilled water (dH_2_O), followed by washed in 2.5% NaOCl (added 2–3 drops of Tween-20) for 15 min and rinsed 3 times with sterilized distilled water (dH_2_O). The stems sterilized pre-cultured in 1/2 MS solid medium and 25 °C in the dark for a week.

### Isolation of CMCs and DDCs

The stem pre-cultured for a week was cut into 1 cm long sections using a sterile scissors, carefully be placed on a sterile Petri dish with filter paper. Cambium, phloem, cortex and epidermal tissue were peeled off from the xylem and the epidermal tissue side was laid on MS (PhytoTechnology Laboratories, USA) medium containing 2 mg L^−1^ 2,4-D, 2 mg L^−1^ NAA, 30 g L^−1^ sucrose and 8 g L^−1^ agar, pH 5.8–6.0, and the isolated tissue were gently pressed to make full contact with the medium. For the observation of lignin, tissues were stained with phloroglucinol-HCl (1.0% (w/v) phloroglucinol (Innochem Technology, China) in 7 mol L^−1^ HCl) for 3 min and then observed under a light microscope (DM6000B, Leica, Germany). After 7 days, DDCs started to form from the layer that consisted of phloem, cortex and epidermis by dedifferentiation. CMCs formed at 30 days post-culture and a visible split was observed between them, which probably due to the discrepancy between cell division rates. CMCs were dim, soft and flat, while DDCs were white, irregular and a bit hard.

### Establishment of cell suspension cultures

Suspension cultures were established by transferring a sample of 2.5 g (fresh cell weight) cells cultured for 12 days on solid medium into 100 mL Erlenmeyer flasks including 40 mL of MS medium containing 2 mg L^−1^ 2,4-D, 2 mg L^−1^ NAA, 30 g L^−1^ sucrose, pH 5.8–6.0, The flasks were agitated at 120 rpm and 25 °C in the dark. Subculturing was undertaken at 3-week intervals.

### Microscopy

Vacuole experimentation was undertaken based on modifications of the methods described previously. CMCs and DDCs were obtained from suspension cultures. CMCs and DDCs were stained with neutral red (C0125, Beyotime Biotechnology, China) for 10 min, then washed with 0.01 mol phosphate buffer (pH 7.2) several times and were observed with a light microscope using the same buffer.

### Response to radiomimetic drug zeocin

CMCs and DDCs suspension cells were treated with zeocin (200 μg mL^−1^, Aladdin, China) at 10 d after subculture, essentially as described previously [22], and then incubated at 25 °C, 120 rpm in the dark for 24 or 48 h. For cell death determination, cells were treated with fluorescein diacetate (200 ng mL^−1^, Sigma, USA) for 20 min and washed with 0.1 mol phosphate buffer several times, then transferred to a microscope slide covered with a thin cover slip, and observed under a fluorescent inverted microscope (Scope A1, Zeiss, Germany). The number of live cells (stained green) were counted. For each sample, five different fields were analyzed and the average cell death rate was measured by excluding the maximum and minimum number of cell counts. All experiments were undertaken in triplicate. Cell death rate = (total number of cells − number of living cells)/total number of cells.

### Sample preparation for AFM

Samples were obtained from suspension CMCs and DDCs at 10 d after subculture to fixation in 2.5% glutaraldehyde for 24 h (volume of cells to liters of solution was 1:10), dehydrated with series-grade ethanol, then embedded with resin, from which 1 μm sections were obtained using an ultramicrotome (UC7, Leica, Germany) and placed on a freshly cleaved mica and dried in air. This enabled cells to adhere to the mica.

### AFM image analysis

Microscope of multimode 8 with NanoScope V controller (Bruker, Germany) and the probe (Snl-10, Bruker, Germany) were utilized for AFM analysis. Imaging was performed at the rate of 1.00 Hz with the contact mode. Surface areas of 20 μm were randomly selected for scanning by the instrument, and ten different areas were scanned for each treatment. The software NanoScope Analysis 1.7 was used AFM image processing and later measurement.

### Sample collection and preparation for transcriptome

RNA from CMCs and DDCs at 10 days after subculture were extracted using the Total RNA Extraction Kit (Promega, Shanghai, China). RNA purity was checked using the NanoPhotometer^®^ spectrophotometer (IMPLEN, CA, USA). RNA concentration was measured using Qubit^®^ RNA Assay Kit in Qubit^®^ 2.0 Fluorometer (Life Technologies, CA, USA) and RNA integrity was assessed using the RNA Nano 6000 Assay Kit of the Agilent Bioanalyzer 2100 system (Agilent Technologies, CA, USA). Each type of cell had 3 biological replicates.

### Library preparation for transcriptome sequencing

Total RNA from all samples was extracted using the Total RNA Extraction Kit (Promega, Shanghai, China). A total amount of 1.5 μg RNA per sample was used as input material for the RNA sample preparations. Sequencing libraries were generated using NEBNext^®^ Ultra™ RNA Library Prep Kit for Illumina^®^ (NEB, USA) following manufacturer’s recommendations and index codes were added to attribute sequences to each sample. Briefly, mRNA was purified from total RNA using poly-T oligo-attached magnetic beads. Fragmentation was carried out using divalent cations under elevated temperature in NEBNext First Strand Synthesis Reaction Buffer (5×). First strand cDNA was synthesized using random hexamer primer and M-MuLV Reverse Transcriptase (RNase H^−^). Second strand cDNA synthesis was subsequently performed using DNA Polymerase I and RNase H. Remaining overhangs were converted into blunt ends via exonuclease/polymerase activities. After adenylation of 3′ ends of DNA fragments, NEBNext Adaptor with hairpin loop structure were ligated to prepare for hybridization. In order to select cDNA fragments of preferentially 150–200 bp in length, the library fragments were purified with AMPure XP system (Beckman Coulter, Beverly, USA). Then 3 μL USER Enzyme (NEB, USA) was used with size-selected, adaptor-ligated cDNA at 37 °C for 15 min followed by 5 min at 95 °C before PCR. Then PCR was performed with Phusion High-Fidelity DNA polymerase, Universal PCR primers and Index Primer. At last, PCR products were purified (AMPure XP system) and library quality was assessed on the Agilent Bioanalyzer 2100 system.

### Transcriptome assembly

The left files from all libraries were pooled into one big left.fq file, and right files into one big right.fq file. Transcriptome assembly was accomplished based on the left.fq and right.fq using Trinity software with min_kmer_cov set to 2 by default and all other parameters set default.

### Clustering and sequencing

The clustering of the index-coded samples was performed on a cBot Cluster Generation System using TruSeq PE Cluster Kit v3-cBot-HS (Illumia) according to the manufacturer’s instructions. After cluster generation, the library preparations were sequenced on an Illumina Hiseq platform and paired-end reads were generated.

### Differential expression analysis

Differential expression analysis of two groups was performed using the DESeq R package. DESeq provide statistical routines for determining differential expression in digital gene expression data using a model based on the negative binomial distribution. The resulting *P* values were adjusted using the Benjamini and Hochberg’s approach for controlling the false discovery rate. Genes with an adjusted P-value < 0.05 found by DESeq were assigned as differentially expressed [52].

### Unigene functional annotation

All unigenes were annotated using BLASTx by searching against seven commonly used databases: the NCBI Nr protein database, NCBI Nt database, Pfam database, KOG database, the Swiss-Prot protein database, GO and KO. The E-values were set as 1e^−5^ (E-value of Pfam was 0.01, *E*-value of KEGG was 10^−10^). GO annotation was carried out using Blast2go and GO enrichment analysis was implemented by the GOseq R packages based Wallenius non-central hyper-geometric distribution, which can adjust for gene length bias in cells [53]. KEGG were used to characterize associated pathways.

### Gene expression analysis

The determination of gene expression levels was carried out by qRT-PCR as previously. Briefly, first-strand cDNA synthesis was done from total RNA from the suspension CMCs and DDCs that were used for the RNA-seq analysis were used as templates for the qRT-PCR analysis. First-strand cDNA was synthesized using the FastQuant RT kit (with gDNase) (Tiangen Biotech, Beijing, China). qRT-PCR using KAPA SYBR FAST qPCR Master Mix Kit (KAPA Biosystems, USA) was performed in the QuantStudio 5 (Thermo Fisher Scientific, USA). Efα gene expression was used as an endogenous control, and the relative expression was analyzed by the 2^−ΔΔCt^ method. The primer sequences employed are listed in Additional file 1: Table S2.

### UPLC/Q-TOF MS

Analysis was undertaken using UPLC/Q-TOF MS (SYNAPT G2-Si, Waters, USA) with an ACQUITY UPLC^®^ HSS T3 chromatographic column (1.8 μm, 2.1 mm × 100 mm, Waters, USA) kept at 40 °C. The method was based on the methods described previously [40]. Briefly, samples (3 μL) were separated using 0.1% (v/v) formic acid (mobile phase A) and 100% acetonitrile (mobile phase B): 0 min at 30% B, 6 min at 45% B, 18 min at 60% B, 23 min at 90% B, at 0.5 mL min^−1^ flow rate. Mass detection was conducted by electrospray ionization (ESI) in the positive ion mode. The QTOF-MS conditions were as follows: sample cone, 40 V; source temperature, 120 °C; desolvation temperature, 450 °C; cone gas flow, 50 L h^−1^; and desolvation gas flow, 800 L/h; capillary voltage, 1000 V. The ramp collision energy was set as 20–65 eV for the high-energy scans. Data analysis was performed using the MassLynx V4.1, Progenesis QI 3.0.3 and EZinfo 3.0 software (Waters). Online database included KEGG, MassBank, Nature Communications, Nature Chemistry, Nature Chemical Biology, and ChemSpider. Variable importance parameter (VIP) value > 1.

### Cells induction with methyl jasmonate

62.5 g L^−1^ (fresh cell weight) cell were cultured in liquid MS medium containing 2 mg L^−1^ 2,4-D, 2 mg L^−1^ NAA, 30 g L^−1^ sucrose. At 10 d after subculture, the elicitor MJ (392707, Sigma, USA) was dissolved in Dimethyl sulfoxide (DMSO, D8418, Sigma, USA), were added into the liquid medium of suspension CMCs and DDCs, making the final concentration of MJ was 50 μmol L^−1^. The control group was added with the same volume of DMSO as MJ, five biological repeats per group. Content of triptolide, celastrol and triptophenolide were measured at 0 h, 12 h, 24 h, 48 h, 120 h, 240 h, 360 h and 480 h.

### Long-term induction with methyl jasmonate

For Long-term induction of culture, CMCs and DDCs were initiated in a similar fashion to that described for the 100 mL Erlenmeyer flask. On day 10, cultures were elicited with 50 μmol L^−1^ and the same volume of DMSO as MJ. After 20 days, aseptically added 50 μmol L^−1^ and MS medium matching cell amount with 2 mg L^−1^ 2,4-D, 2 mg L^−1^NAA, 30 g L^−1^ sucrose as same initial inoculum content of 62.5 mg mL^−1^ every 20 days. After 6 months of continuous culture, intracellular and extracellular terpenoids levels were analyzed.

### Analysis of triptolide, celastrol and triptophenolide content

After cells were separated from the medium, cells were freeze-dried *in vacuo* for 48 h, growth of them determined as DW (g). 65 mg of cells were weighed and then soaked in 1.5 mL 80% (v/v) methanol for 12 h, followed by 40 kHz ultrasonication at 25 °C for 1 h. The methanol extracts were centrifuged at 13,000 rpm for 10 min and the supernatant were filtered through a 0.22 μm microporous membrane. UPLC (1290 Infinity II, Agilent) with an ACQUITY UPLC^®^ HSS T3 chromatographic column (1.8 μm, 2.1 mm × 100 mm, Waters) was used for the analysis. Column temperature was 40 °C and the flow rate was 0.4 mL min^−1^. 100% acetonitrile (mobile phase A) and 0.1% (v/v) formic acid in water (mobile phase B) with a linear gradient was used: (0 min: 70% B, 5 min: 65% B, 15 min: 30% B, 21 min: 10% B). A UV detector monitored at 219.4 nm, 260.0 and 426.0 nm, the sample injection volume was 5 μL. For determining the extracellular triptolide, celastrol and triptophenolide concentration, medium (25 mL) was extracted 3 times with the same volume of ethyl acetate. The combined ethyl acetate fraction was subsequently concentrated and then redissolved in 1 mL 80% methanol and was filtered through a 0.22 μm microporous membrane before UPLC analysis as previously described.

## Supplementary information

**Additional file 1: Figure S1.** The isolation of cambium cell layer from xylem tissue of *T. wilfordii*. **Figure S2.** Transcriptome data of CMCs and DDCs from *T. wilfordii*. **Figure S3.** Amino acid sequence alignment between *T. wilfordii* cluster 96, cluster 95, cluster 71, cluster 04 and CMC marker genes. **Figure S4.** UPLC-QTOF-MS traces (total ion current) obtained with methanolic extracts of CMCs and DDCs. **Figure S5.** MS spectra of compound 1 from the [M + H]^+^ ion at m/z 361.11. **Figure S6.** MS spectra of compound 2 from the [M + H]^+^ ion at m/z 313.17. **Figure S7.** MS spectra of compound 3 from the [M + H]^+^ ion at m/z 451.28. **Figure S8.** Terpenoids production of *T. wilfordii* DDCs following induction of 50 μmol L^−1^ MJ. **Figure S9.** Effect of 50 μmol L^−1^-MJ on growth of *T. wilfordii* DDCs. **Table S1.** Length distribution of transcriptional data. **Table S2.** The primers used for qRT-PCR. **Table S3.** Related data of target compounds detected by UPLC/Q-TOF MS.

## Data Availability

All data generated or analyzed during this study are available in this published article and its additional files.

## Abbreviations

2,4-D: 2,4-dichlorophenoxyacetic acid
CMCs: cambial meristematic cells
CLE: CLAVATA3/EMBRYO SURROUNDING REGION-RELATED
CK: control check
DDCs: dedifferentiated cells
DEGs: differentially expressed genes
DW: dry weight
KOG: eukaryotic ortholog groups
GO: gene ontology
GA: gibberellic acid
IPP: isopentenyl pyrophosphate
KEGG: Kyoto Encyclopedia of Genes and Genomes
KO: Kyoto Encyclopedia of Genes and Genomes Orthology
RLK: leucine-rich repeat receptor-like kinase
MJ: methyl jasmonate
NCBI Nr: National Center for Biotechnology Information non-redundant
NAA: naphthalene-acetic acid
Nt: National Center for Biotechnology Information nucleotide
OPLS-DA: orthogonal partial least square-discriminant analysis
*PXY*: *phloem intercalated with xylem*
Pfam: protein family
qRT-PCR: quantitative real-time reverse transcription-polymerase chain reaction
RNA-seq: RNA-sequencing
*TwDXS*: *Tripterygium wilfordii 1*-*deoxy*-d-*xylulose*-*5*-*phosphate synthase*
*TwDXR*: *Tripterygium wilfordii 1*-*deoxy*-d-*xylulose*-*5*-*phosphate reductoisomerase*
*TwHMGS*: *Tripterygium wilfordii hydroxymethylglutaryl*-*CoA synthase*
*TwHMGR*: *Tripterygium wilfordii hydroxymethylglutaryl*-*CoA reductase*
*TwIDI*: *Tripterygium wilfordii isopentenyl diphosphate isomerase*
*TwGGPS*: *Tripterygium wilfordii geranylgeranyl diphosphate synthase*
*TwFPS*: *Tripterygium wilfordii farnesyl diphosphate synthase*
UPLC: ultra-performance liquid chromatography
UPLC/Q-TOF MS: ultra-performance liquid chromatography-quadrupole time-of-flight mass spectrometry
*WUS*: *WUSCHEL*

## References

[1] Liu CZ, Zhao Y, Wang YC (2006). Artemisinin: current state and perspectives for biotechnological production of an antimalarial drug. Appl Microbiol Biotechnol.

[2] Fischer R, Vasilev N, Twyman RM, Schillberg S (2015). High-value products from plants: the challenges of process optimization. Curr Opin Biotechnol.

[3] Zheng L, Fu Y, Zhuang L, Gai R, Ma J, Lou J, Zhu H, He Q, Yang B (2014). Simultaneous NF-kappaB inhibition and E-cadherin upregulation mediate mutually synergistic anticancer activity of celastrol and SAHA in vitro and in vivo. Int J Cancer.

[4] Wang X, Liang XB, Li FQ, Zhou HF, Liu XY, Wang JJ, Wang XM (2008). Therapeutic strategies for Parkinson’s disease: the ancient meets the future-traditional Chinese herbal medicine, electroacupuncture, gene therapy and stem cells. Neurochem Res.

[5] Chen YW, Lin GJ, Chia WT, Lin CK, Chuang YP, Sytwu HK (2009). Triptolide exerts anti-tumor effect on oral cancer and KB cells in vitro and in vivo. Oral Oncol.

[6] Guo X, Xue M, Li CJ, Yang W, Wang SS, Ma ZJ, Zhang XN, Wang XY, Zhao R, Chang BC (2016). Protective effects of triptolide on TLR4 mediated autoimmune and inflammatory response induced myocardial fibrosis in diabetic cardiomyopathy. J Ethnopharmacol.

[7] Zhang LY, Li H, Wu YW, Cheng L, Yan YX, Yang XQ, Zhu FH, He SJ, Tang W, Zuo JP (2017). (5R)-5-hydroxytriptolide ameliorates lupus nephritis in MRL/lpr mice by preventing infiltration of immune cells. Am J Physiol Renal Physiol.

[8] Reno TA, Kim JY, Raz DJ (2015). Triptolide inhibits lung cancer cell migration, invasion, and metastasis. Ann Thorac Surg..

[9] Liu XH, Zhao PY, Wang XJ, Wang L, Zhu YJ, Gao W (2019). Triptolide induces glioma cell autophagy and apoptosis via upregulating the ROS/JNK and downregulating the Akt/mTOR signaling pathways. Front Oncol..

[10] Meng C, Zhu H, Song H, Wang Z, Huang G, Li D, Ma Z, Ma J, Qin Q, Sun X (2014). Targets and molecular mechanisms of triptolide in cancer therapy. Chin J Cancer Res.

[11] Liu J, Lee J, Salazar Hernandez MA, Mazitschek R, Ozcan U (2015). Treatment of obesity with celastrol. Cell.

[12] Liu XH, Zhao PY, Wang XJ, Wang L, Zhu YJ, Song YD, Gao W (2019). Celastrol mediates autophagy and apoptosis via the ROS/JNK and Akt/mTOR signaling pathways in glioma cells. J Exp Clin Cancer Res..

[13] He Y, Wu M, Liu Y, Li Q, Li X, Hu L, Cen S, Zhou J (2016). Identification of triptophenolide from *Tripterygium wilfordii* as a pan-antagonist of androgen receptor. ACS Med Chem Lett..

[14] Corson TW, Crews CM (2007). Molecular understanding and modern application of traditional medicines: triumphs and trials. Cell.

[15] Guo L, Duan L, Liu K, Liu EH, Li P (2014). Chemical comparison of *Tripterygium wilfordii* and *Tripterygium hypoglaucum* based on quantitative analysis and chemometrics methods. J Pharm Biomed Anal.

[16] Nakano K, Yoshida C, Furukawa W, Takaishi Y, Shishido K (1998). Terpenoids in transformed root culture of *Tripterygium wilfordii*. Phytochemistry.

[17] Zhu C, Miao G, Guo J, Huo Y, Zhang X, Xie J, Feng J (2014). Establishment of *Tripterygium wilfordii* Hook. f. hairy root culture and optimization of its culture conditions for the production of triptolide and wilforine. J Microbiol Biotechnol..

[18] Miao GP, Zhu CS, Yang YQ, Feng MX, Ma ZQ, Feng JT, Zhang X (2014). Elicitation and in situ adsorption enhanced secondary metabolites production of *Tripterygium wilfordii* Hook. f. adventitious root fragment liquid cultures in shake flask and a modified bubble column bioreactor. Bioprocess Biosyst Eng..

[19] Thorpe T (2012). History of plant tissue culture. Methods Mol Biol.

[20] Roberts S, Kolewe M (2010). Plant natural products from cultured multipotent cells. Nat Biotechnol.

[21] Roberts SC (2007). Production and engineering of terpenoids in plant cell culture. Nat Chem Biol.

[22] Lee EK, Jin YW, Park JH, Yoo YM, Hong SM, Amir R, Yan Z, Kwon E, Elfick A, Tomlinson S (2010). Cultured cambial meristematic cells as a source of plant natural products. Nat Biotechnol.

[23] Laux T (2003). The stem cell concept in plants: a matter of debate. Cell.

[24] Ye ZH (2002). Vascular tissue differentiation and pattern formation in plants. Annu Rev Plant Biol.

[25] Lee SB, Cho HI, Jin YW, Lee EK, Ahn JY, Lee SM (2016). Wild ginseng cambial meristematic cells ameliorate hepatic steatosis and mitochondrial dysfunction in high-fat diet-fed mice. J Pharm Pharmacol.

[26] Zhang Y, Jiang K, Qing D, Huang B, Jiang J, Wang S, Yan C (2017). Accumulation of camptothecin and 10-hydroxycamptothecin and the transcriptional expression of camptothecin biosynthetic genes in *Camptotheca acuminata* cambial meristematic and dedifferentiated cells. RSC Advances..

[27] Moon SH, Venkatesh J, Yu JW, Park SW (2015). Differential induction of meristematic stem cells of *Catharanthus roseus* and their characterization. C R Biol..

[28] Frankenstein C, Eckstein D, Schmitt U (2005). The onset of cambium activity-A matter of agreement?. Dendrochronologia..

[29] Joshi JB, Elias CB, Patole MS (1996). Role of hydrodynamic shear in the cultivation of animal, plant and microbial cells. Chem Eng J Biochem Eng J..

[30] Grabherr MG, Haas BJ, Yassour M, Levin JZ, Thompson DA, Amit I, Adiconis X, Fan L, Raychowdhury R, Zeng Q (2011). Full-length transcriptome assembly from RNA-Seq data without a reference genome. Nat Biotechnol.

[31] Fisher K, Turner S (2007). PXY, a receptor-like kinase essential for maintaining polarity during plant vascular-tissue development. Curr Biol.

[32] Yadav RK, Perales M, Gruel J, Girke T, Jonsson H, Reddy GV (2011). WUSCHEL protein movement mediates stem cell homeostasis in the *Arabidopsis* shoot apex. Genes Dev.

[33] Schoof H, Lenhard M, Haecker A, Mayer KF, Jurgens G, Laux T (2000). The stem cell population of *Arabidopsis* shoot meristems in maintained by a regulatory loop between the *CLAVATA* and *WUSCHEL* genes. Cell.

[34] Sakamoto T, Kamiya N, Ueguchi-Tanaka M, Iwahori S, Matsuoka M (2001). KNOX homeodomain protein directly suppresses the expression of a gibberellin biosynthetic gene in the tobacco shoot apical meristem. Genes Dev.

[35] Vranova E, Coman D, Gruissem W (2013). Network analysis of the MVA and MEP pathways for isoprenoid synthesis. Annu Rev Plant Biol.

[36] Zhao YJ, Zhang YF, Su P, Yang J, Huang LQ, Gao W (2017). Genetic Transformation system for woody plant *Tripterygium wilfordii* and its application to product natural celastrol. Front Plant Sci..

[37] Bylesjö M, Rantalainen M, Cloarec O, Nicholson JK, Holmes E, Trygg J (2006). OPLS discriminant analysis: combining the strengths of PLS-DA and SIMCA classification. J Chemom.

[38] Zeng F, Wang W, Guan S, Cheng C, Yang M, Avula B, Khan IA, Guo DA (2013). Simultaneous quantification of 18 bioactive constituents in *Tripterygium wilfordii* using liquid chromatography-electrospray ionization-mass spectrometry. Planta Med.

[39] Guo H, Wang Z, Xu L, Zhang H, Chang R, Chen A (2019). Separation and simultaneous determination of seven bioactive components in *Tripterygium wilfordii* Hook. F. and *Tripterygium* preparations by micellar electrokinetic capillary chromatography. Electrophoresis..

[40] Su P, Guan HT, Zhao YJ, Tong YR, Xu MM, Zhang YF, Hu TY, Yang J, Cheng QQ, Gao LH (2018). Identification and functional characterization of diterpene synthases for triptolide biosynthesis from *Tripterygium wilfordii*. Plant J..

[41] Zhou JW, Hu TY, Gao LH, Su P, Zhang YF, Zhao YJ, Chen S, Tu LC, Song YD, Wang X (2019). Friedelane-type triterpene cyclase in celastrol biosynthesis from *Tripterygium wilfordii* and its application for triterpenes biosynthesis in yeast. New Phytol.

[42] Tong YR, Su P, Zhao YJ, Zhang M, Wang X, Liu YJ, Zhang XN, Gao W, Huang LQ (2015). Molecular cloning and characterization of *DXS* and *DXR* genes in the terpenoid biosynthetic pathway of *Tripterygium wilfordii*. Int J Mol Sci.

[43] Kochan E, Szymczyk P, Kuzma L, Szymanska G, Wajs-Bonikowska A, Bonikowski R, Sienkiewicz M (2018). The increase of triterpene saponin production induced by trans-anethole in hairy root cultures of *Panax quinquefolium*. Molecules..

[44] Hao XL, Shi M, Cui LJ, Xu C, Zhang YJ, Kai GY (2015). Effects of methyl jasmonate and salicylic acid on tanshinone production and biosynthetic gene expression in transgenic *Salvia miltiorrhiza* hairy roots. Biotechnol Appl Biochem.

[45] Rodriguez CM, Boronat A (2002). Elucidation of the methylerythritol phosphate pathway for isoprenoid biosynthesis in bacteria and plastids. A metabolic milestone achieved through genomics. Plant Physiol..

[46] Liu YJ, Zhao YJ, Zhang M, Su P, Wang XJ, Zhang XN, Gao W, Huang LQ (2014). Cloning and characterisation of the gene encoding 3-hydroxy-3-methylglutaryl-CoA synthase in *Tripterygium wilfordii*. Molecules.

[47] Singh S, Pal S, Shanker K, Chanotiya CS, Gupta MM, Dwivedi UN, Shasany AK (2015). Sterol partitioning by *HMGR* and *DXR* for routing intermediates toward withanolide biosynthesis. Physiol Plant.

[48] Shi M, Luo XQ, Ju GH, Li LL, Huang SX, Zhang T, Wang HZ, Kai GY (2016). Enhanced diterpene tanshinone accumulation and bioactivity of transgenic *Salvia miltiorrhiza* hairy roots by pathway engineering. J Agric Food Chem.

[49] Zhou W, Huang F, Li S, Wang Y, Zhou C, Shi M, Wang J, Chen Y, Wang Y, Wang H (2016). Molecular cloning and characterization of two 1-deoxy-d-xylulose-5-phosphate synthase genes involved in tanshinone biosynthesis in *Salvia miltiorrhiza*. Mol Breeding.

[50] Su P, Tong YR, Cheng QQ, Hu YT, Zhang M, Yang J, Teng ZQ, Gao W, Huang LQ (2016). Functional characterization of ent-copalyl diphosphate synthase, kaurene synthase and kaurene oxidase in the *Salvia miltiorrhiza* gibberellin biosynthetic pathway. Sci Rep..

[51] Zhou YJ, Gao W, Rong Q, Jin G, Chu H, Liu W, Yang W, Zhu Z, Li G, Zhu G (2012). Modular pathway engineering of diterpenoid synthases and the mevalonic acid pathway for miltiradiene production. J Am Chem Soc.

[52] Wang L, Feng Z, Wang X, Wang X, Zhang X (2010). DEGseq: an R package for identifying differentially expressed genes from RNA-seq data. Bioinformatics.

[53] Young MD, Wakefield MJ, Smyth GK, Oshlack A (2010). Gene ontology analysis for RNA-seq: accounting for selection bias. Genome Biol.

