# Supplementary material for: A novel strategy to enhance terpenoids production using cambial meristematic cells of Tripterygium wilfordii Hook. f
Source: Plant Methods. 2019 Nov 7;15:129. doi: 10.1186/s13007-019-0513-x (PMC6836502; doi:10.1186/s13007-019-0513-x)
Supplement: Supplementary file 1 — Additional file 1: Figure S1. The isolation of cambium cell layer from xylem tissue of T. wilfordii. Figure S2. Transcriptome data of CMCs and DDCs from T. wilfordii. Figure S3. Amino acid sequence alignment between T. wilfordii cluster 96, cluster 95, cluster 71, cluster 04 and CMC marker genes. Figure S4. UPLC-QTOF-MS traces (total ion current) obtained with methanolic extracts of CMCs and DDCs. Figure S5. MS spectra of compound 1 from the [M + H]+ ion at m/z 361.11. Figure S6. MS spectra of compound 2 from the [M + H]+ ion at m/z 313.17. Figure S7. MS spectra of compound 3 from the [M + H]+ ion at m/z 451.28. Figure S8. Terpenoids production of T. wilfordii DDCs following induction of 50 μmol L−1 MJ. Figure S9. Effect of 50 μmol L−1-MJ on growth of T. wilfordii DDCs. Table S1. Length distribution of transcriptional data. Table S2. The primers used for qRT-PCR. Table S3. Related data of target compounds detected by UPLC/Q-TOF MS. [file 13007_2019_513_MOESM1_ESM.docx]

**Additional Material**

**A novel strategy to enhance terpenoids production using cambial meristematic cells of *Tripterygium wilfordii* Hook. f.**

**Yadi Song^1^, Shang Chen^1^, Xiujuan Wang^1*^, Rui Zhang^1^, Lichan Tu^1^, Tianyuan Hu^1^, Xihong Liu^1^, Yifeng Zhang^1^, Luqi Huang^3^, Wei Gao^1,2,4*^**

^1^ School of Traditional Chinese Medicine, Capital Medical University, Beijing, 100069, China

^2^ School of Pharmaceutical Sciences, Capital Medical University, Beijing, 100069, China

^3^ State Key Laboratory of Dao-di Herbs, National Resource Center for Chinese Materia Medica, China Academy of Chinese Medical Sciences, Beijing, 100700, China

^4^Advanced Innovation Center for Human Brain Protection, Capital Medical University, Beijing, 100069, China

*Corresponding authors:

Xiujuan Wang: [wxj0517@sina.com](mailto:wxj0517@sina.com);

Wei Gao: [weigao@ccmu.edu.cn](mailto:weigao@ccmu.edu.cn).

**CONTENT**

- Figure S1 The isolation of cambium cell layer from xylem tissue of *T. wilfordii*;

- Figure S2 Transcriptome data of CMCs and DDCs from *T. wilfordii*;

- Figure S3 Amino acid sequence alignment between *T. wilfordii* cluster 96, cluster 95, cluster 71, cluster 04 and CMC marker genes;

- Figure S4 UPLC-QTOF-MS traces (total ion current) obtained with methanolic extracts of CMCs and DDCs.

- Figure S5 MS spectra of compound 1 from the [M+H]^+^ ion at m/z 361.11;

- Figure S6 MS spectra of compound 2 from the [M+H]^+^ ion at m/z 313.17;

- Figure S7 MS spectra of compound 3 from the [M+H]^+^ ion at m/z 451.28;

- Figure S8 Terpenoids production of *T. wilfordii* DDCs following induction of 50 μmol L^-1^ MJ;

- Figure S9 Effect of 50 μmol L^-1^-MJ on growth of *T. wilfordii* DDCs;

- Table S1 Length distribution of transcriptional data;

- Table S2 The primers used for qRT-PCR;

- Table S3 Related data of target compounds detected by UPLC/Q-TOF MS.

**
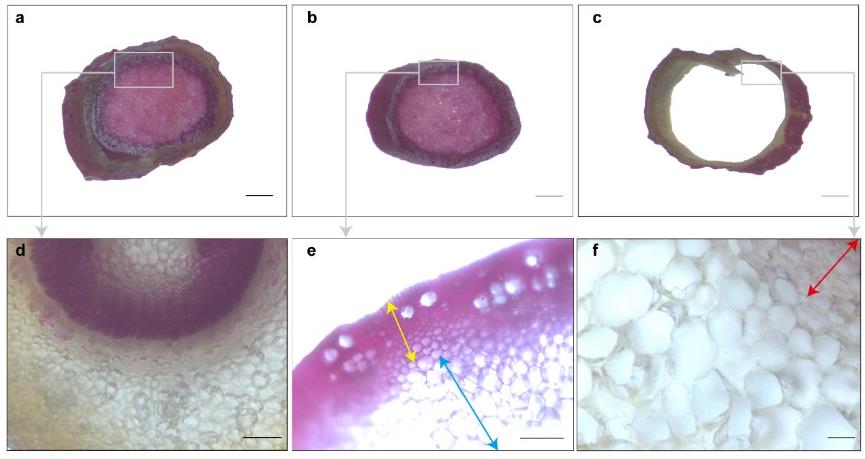
**

**Figure S1** The isolation of cambium cell layer from xylem tissue of *T. wilfordii*. (a) The stem cross-sections of *T. wilfordii* were stained with phloroglucinol-HCl to detect lignin deposition (dyed in purplish-red). (b) Separated xylem and pith tissue from stem segment in panel a, stained with lignin-specific dye, phloroglucinol-HCl. (c) Cambium, phloem, cortex and epidermal tissue were peeled off from the xylem and pith tissue, from stem segment in panel a, stained with lignin-specific dye phloroglucinol-HCl. It was shown that phloroglucinol-HCl did not stain this tissue except epidermis. Xylem tissue was completely separated. (d) Optical microscopic observation of panel a. (e) Optical microscopic observation of panel b. The lignin-specific dye, phloroglucinol-HCl, stained these cells purplish-red. Yellow and blue arrow indicate xylem cell and pith cell layers, respectively. (f) Cross-section of cambium cell layer and phloem tissue from panel c. It was shown that phloroglucinol-HCl did not stain these cells. Red arrow bar indicates cambium cell layers. Scale bar is equivalent to 0.15 mm for a-c, 25 μm for d, 15 μm for e, and 10 μm for f.

**
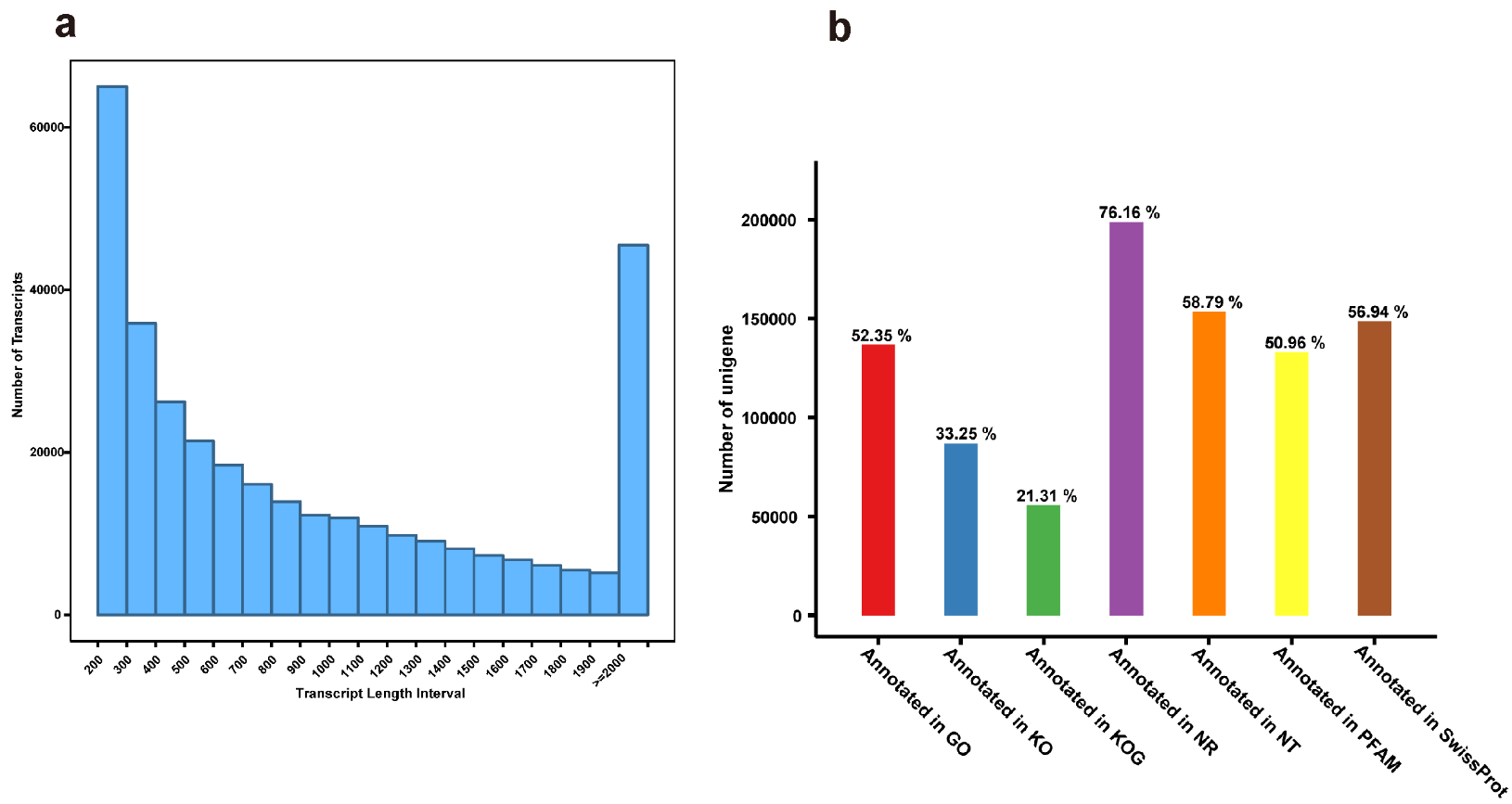
**

**Figure S2** Transcriptome data of CMCs and DDCs from *T. wilfordii*. (a) Transcript length distribution. (b) Success rate of gene annotation.

**
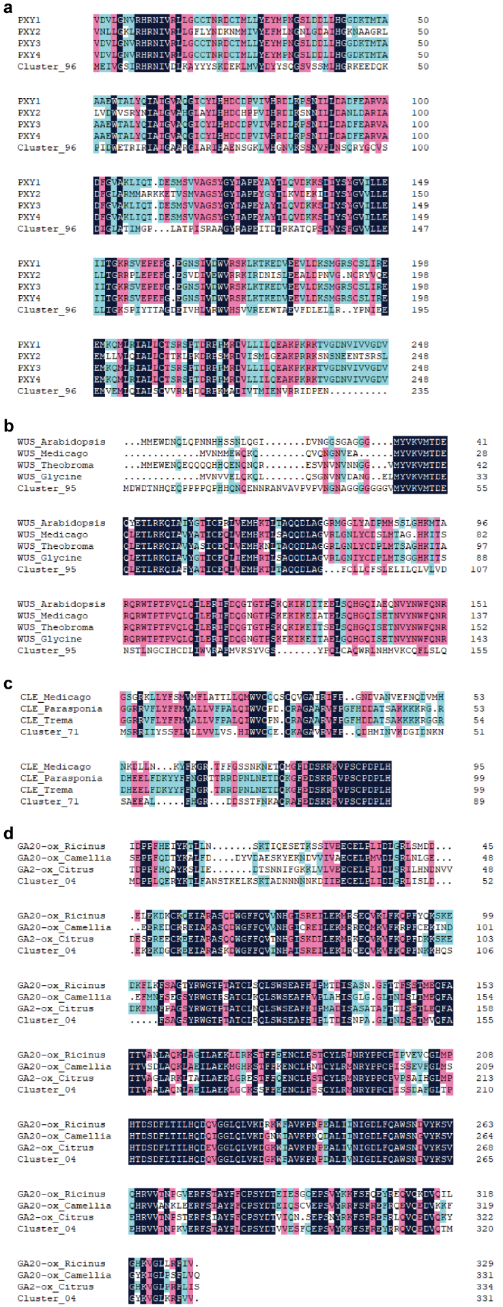
**

**Figure S3** Amino acid sequence alignment between *T. wilfordii* cluster 96, cluster 95, cluster 71, cluster 04 and CMC marker genes. (a) Comparison of *T. wilfordii* cluster 96 and PXY. GenBank ID: PXY1 (NP_200956), PXY2 (NP_194594), PXY3 (AED97473), PXY4 (ACN59400). (b) Comparison of *T. wilfordii* cluster 95 and WUS. GenBank ID: WUS_Arabidopsis (*Arabidopsis thaliana*; NP_195280), WUS_Medicago (*Medicago truncatula*; KEH44470), WUS_Theobroma (*Theobroma cacao*; EOX90656), WUS_Glycine (*Glycine soja*; KHN37332). (c) Comparison of *T. wilfordii* cluster 71 and CLV3 of CLE family. GenBank ID: CLE_Medicago (*Medicago truncatula*; KEH44216), CLE_Parasponia (*Parasponia andersonii*; PON78607), CLE_Trema (*Trema orientalis*; PON95933). (d) Comparison of *T. wilfordii* cluster 04 and GA20-oxidase (GA20-ox). GenBank ID: GA20-ox_Ricinus (*Ricinus communis*; EEF43925), GA20-ox_Camellia (*Camellia sinensis*; AUD40403), GA20-ox_Citrus (*Citrus unshiu*; BAU98538). Sequences were aligned by the multiple sequence alignment programme.

**
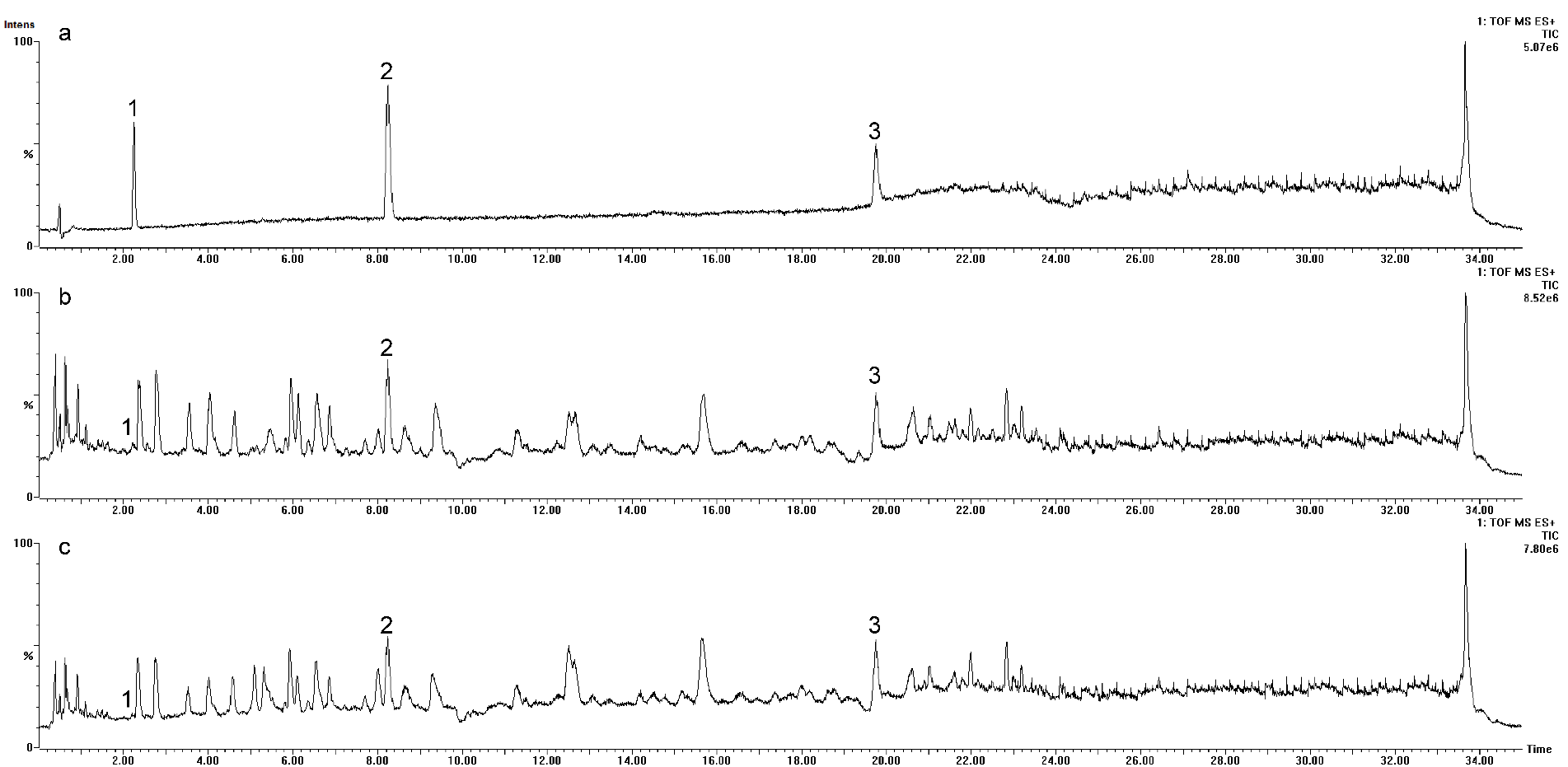
**

**Figure S4** UPLC-QTOF-MS traces (total ion current) obtained with methanolic extracts of CMCs and DDCs. (a) UPLC-UV chromatogram of terpenoid standards. (b) UPLC-UV chromatogram of CMCs. (c) UPLC-UV chromatogram of DDCs. The characterization of the compound peaks 1 to 3 are given in Table S3.


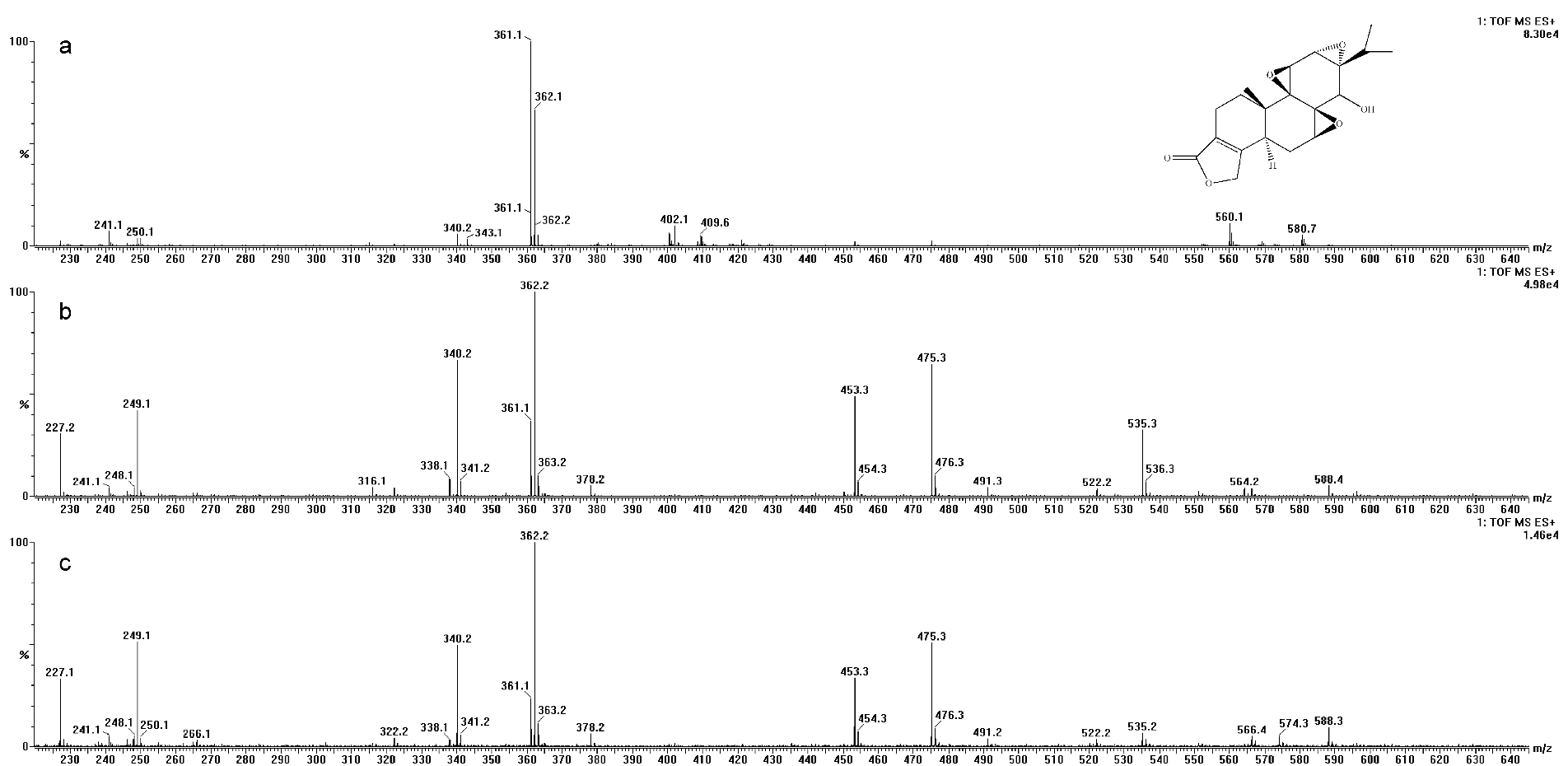


**Figure S5** MS spectra of compound 1 from the [M+H]^+^ ion at m/z 361.11. (a) MS spectrum of terpenoid standards. (b) MS spectrum of CMCs. (c) MS spectrum of DDCs.


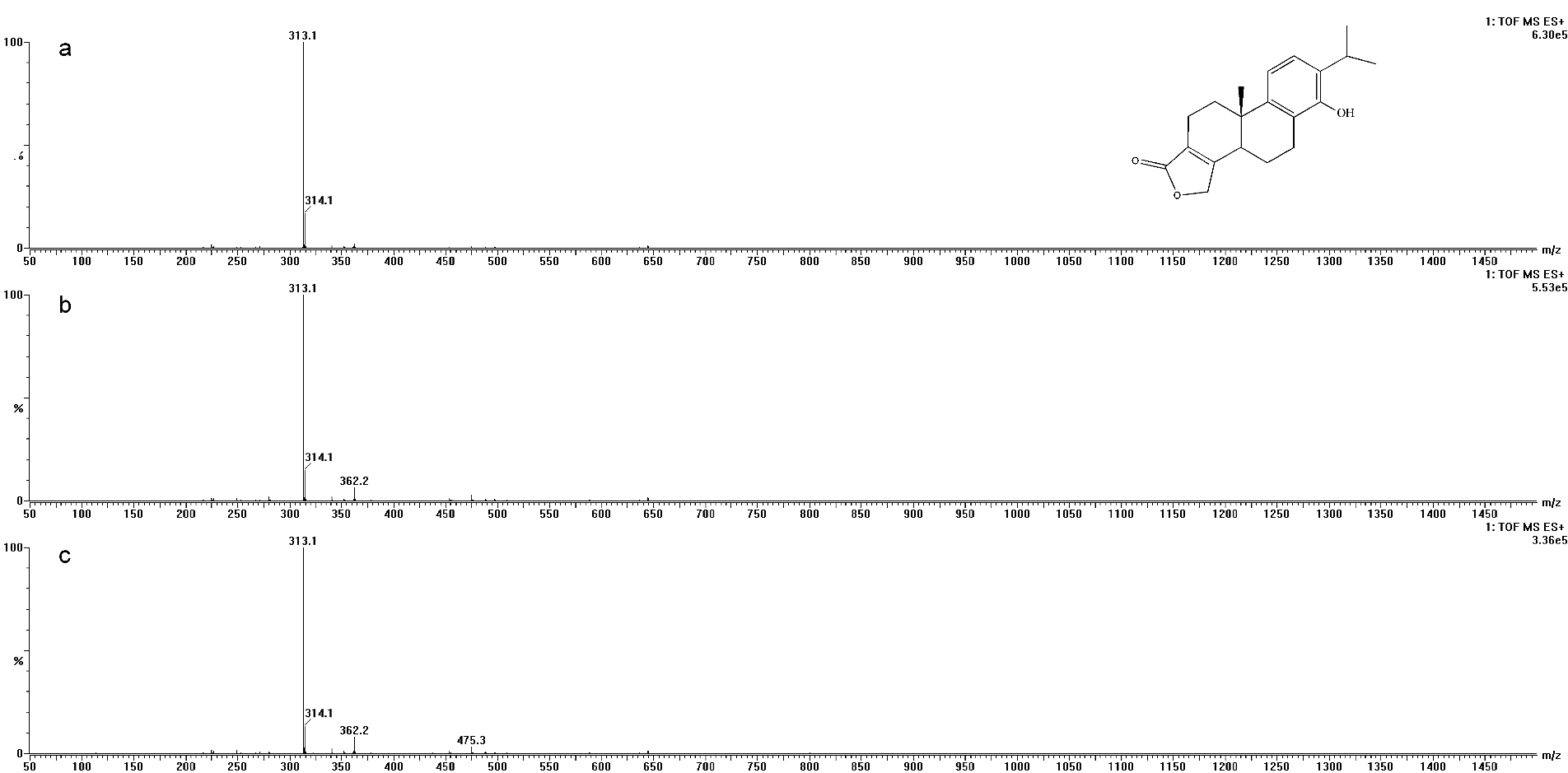


**Figure S6** MS spectra of compound 2 from the [M+H]^+^ ion at m/z 313.17. (a) MS spectrum of terpenoid standards. (b) MS spectrum of CMCs. (c) MS spectrum of DDCs.


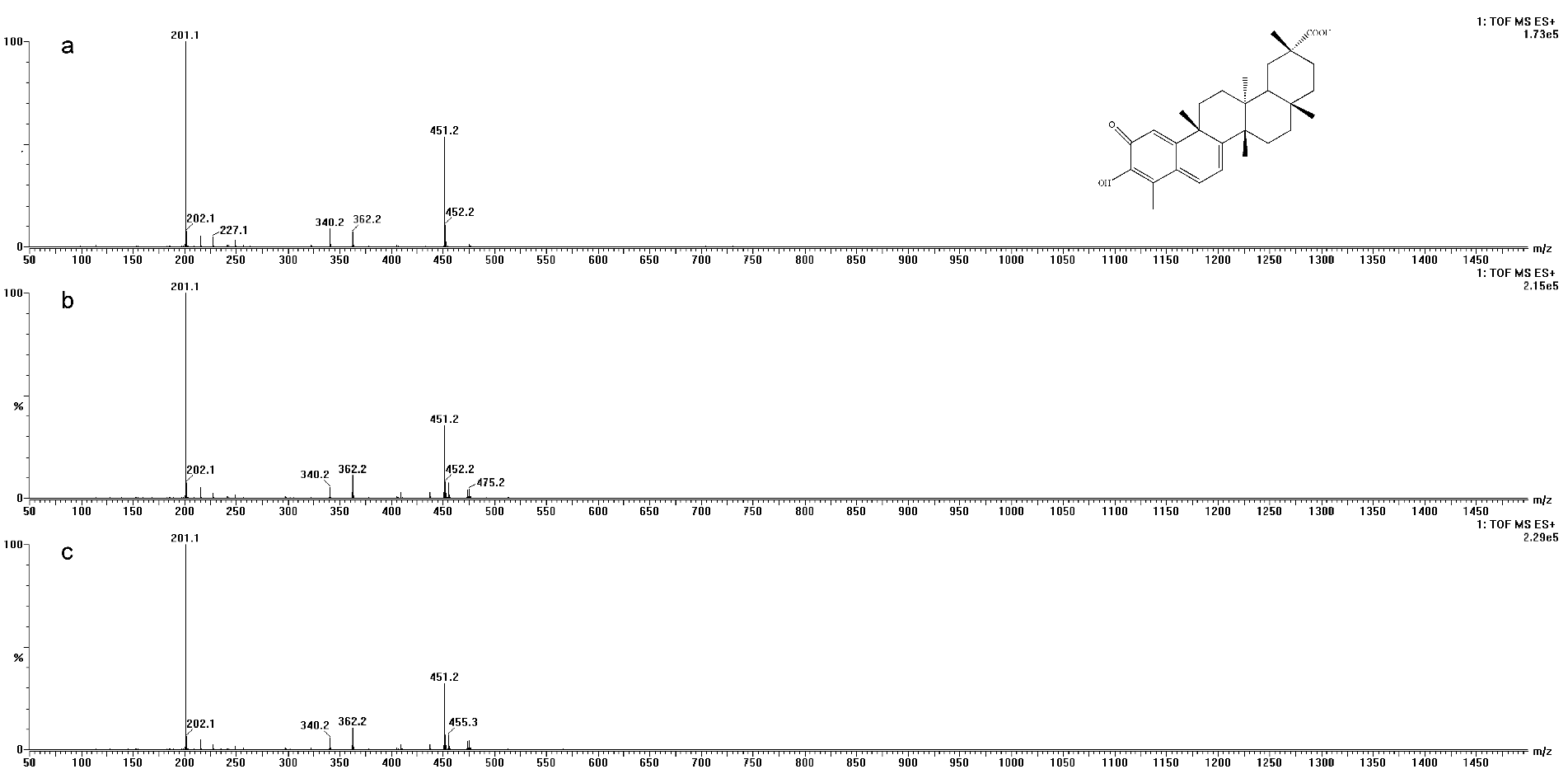


**Figure S7** MS spectra of compound 3 from the [M+H]^+^ ion at m/z 451.28. (a) MS spectrum of terpenoid standards. (b) MS spectrum of CMCs. (c) MS spectrum of DDCs.


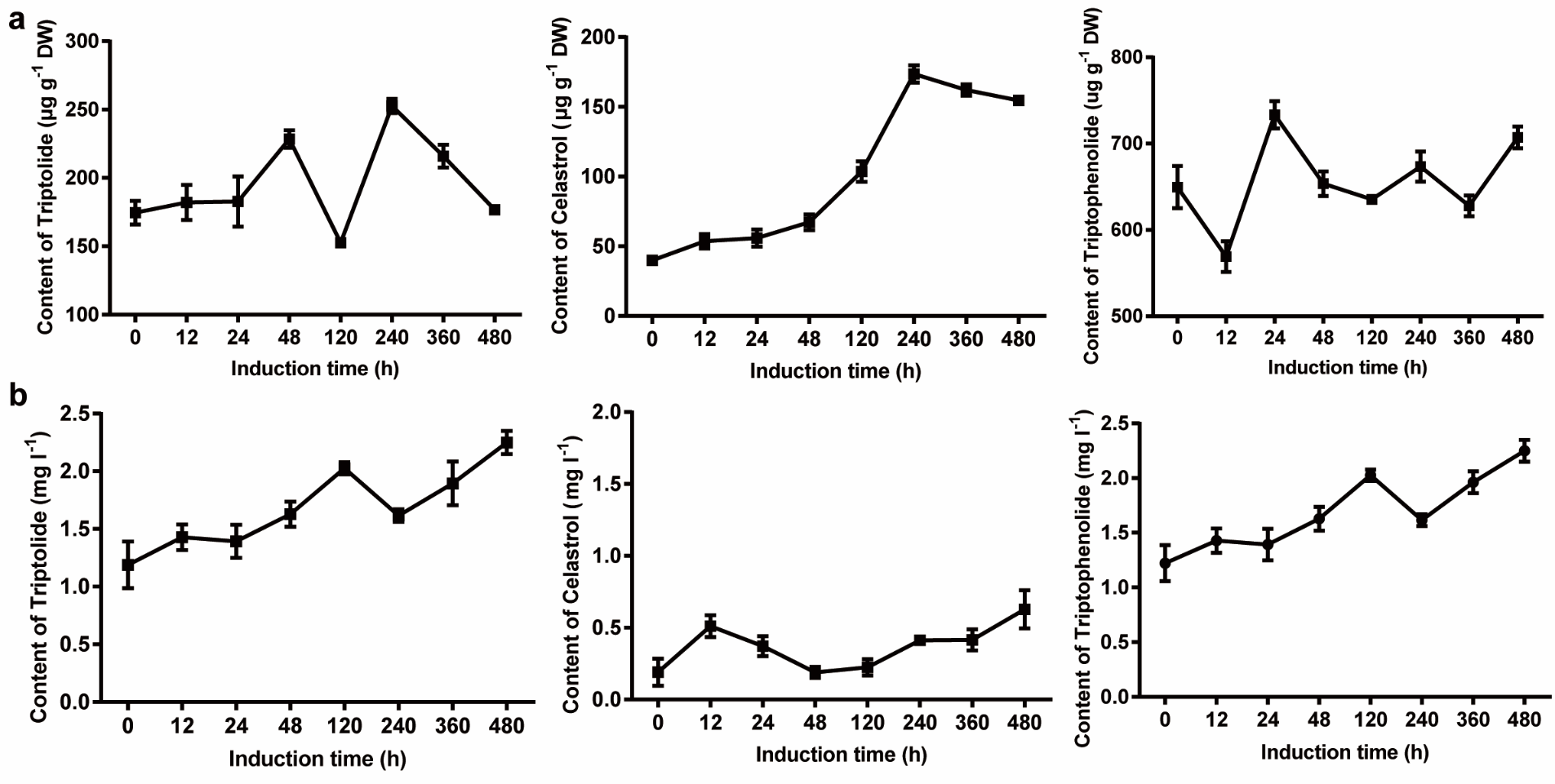


**Figure S8** Terpenoids production of *T. wilfordii* DDCs following induction of 50 μmol L^-1^ MJ. (a) Total triptolide, celastrol and triptophenolide production in cells. (b) Total triptolide, celastrol and triptophenolide production in medium. The data represent the mean ± SD of five independent suspension cell cultures. DW, dry weight.


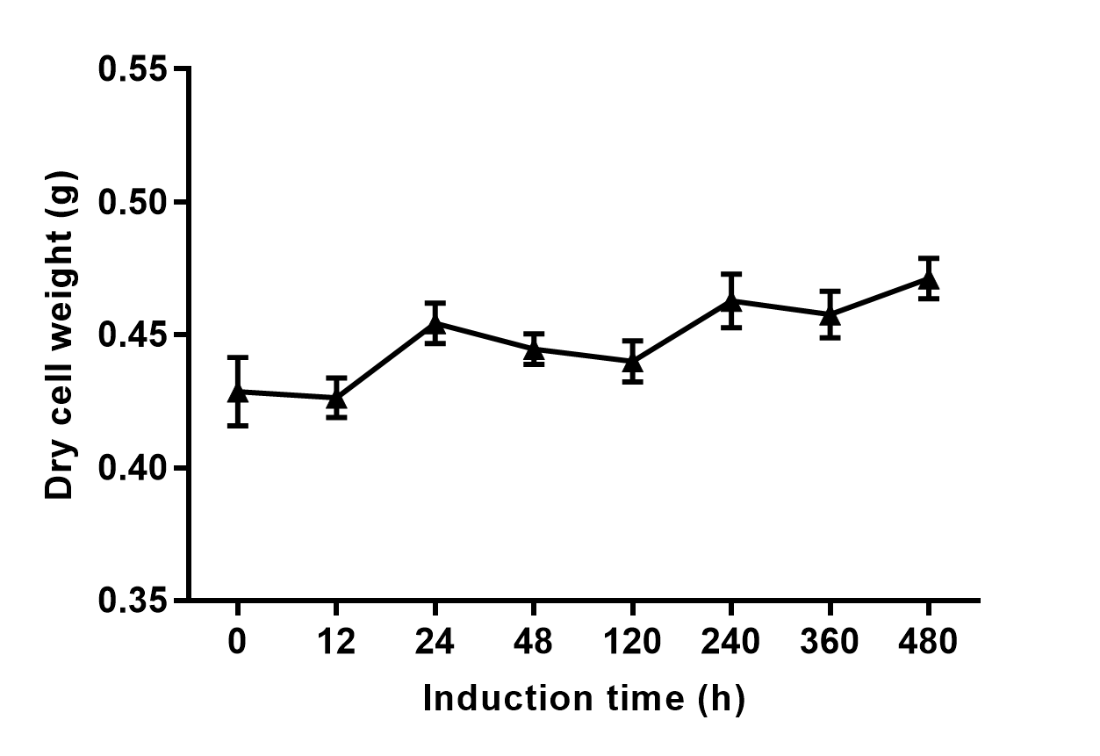


**Figure S9** Effect of 50 μmol L^-1^-MJ on growth of *T. wilfordii* DDCs. Values represent means ± SD, n=5.

**Table S1** Length distribution of transcriptional data

|  | Min  length | Mean length | Median length | Max  length | N50 | N90 |
| --- | --- | --- | --- | --- | --- | --- |
| Transcripts | 201 | 1033 | 704 | 17305 | 1611 | 453 |
| Unigenes | 201 | 1241 | 961 | 17305 | 1712 | 613 |

**Table S2** The primers used for qRT-PCR

| Primer name | Primer Sequence (5'-3') |
| --- | --- |
| Cluster 96-F | TTACAGTCAAGGGAGCGTCTC |
| Cluster 96-R | AGCCATACCGTTGGGAGTT |
| Cluster 95-F | AACCGATGGACTGGGACAC |
| Cluster 95-R | GGAGACAGAAACCTGCGAGA |
| Cluster 71-F | GTCTGCTGCGAGTGTAAGGC |
| Cluster 71-R | GGATCTGGACAACTGGGAACT |
| Cluster 04-F | ATTTGGGCTGACACCACATAC |
| Cluster 04-R | CTGCGAACCATTTACCATCTT |
| qEfα-F | CCAAGGGTGAAAGCAAGGAGAGC |
| qEfα-R | CACTGGTGGTTTTGAGGCTGGTATCT |
| qDXR-F | AATCTCCTTGTTCGGTTCCA |
| qDXR-R | GCTGGTTGAGGCTGCTGA |
| qDXS-F | GGCGACTACTGGGTCTTTCTT |
| qDXS-R | TGTCTTTGCGTATCATCATCCT |
| qGGPS-F | GGCAAGAGGGTTCGTCCAG |
| qGGPS-R | AAAGTAGTGCATCACCAGCAAG |
| qHMGS-F | CTGGAGGTAGGGAGCGAGAC |
| qHMGS-R | CCATAGCAGGCATTGGTTGA |
| qHMGR-F | GCCTTTGCTGCTGGACGACTA |
| qHMGR-R | CCGCTGCTCTTCTGGCTGAC |
| qFPS-F | CAGACCCTCACCTTCCATT |
| qFPS-R | AAGAGTAACCATAAGCAGCAGAC |
| qIDI-F | GTCCCTTCCACCCTAACC |
| qIDI-R | GCCCAACCACACGATCATTC |

**Table S3** Related data of target compounds detected by UPLC/Q-TOF MS

| Number | Retention time  (min) | [M+H]^+^ (m/z) | UV (nm) | Formula | Compound |
| --- | --- | --- | --- | --- | --- |
| 1 | 2.25 | 361.21 | 220 | C_20_H_24_O_6_ | Triptolide |
| 2 | 8.24 | 313.17 | 200 | C_20_H_24_O_3_ | Triptophenolide |
| 3 | 19.75 | 451.28 | 425 | C_29_H_38_O_4_ | Celastrol |
